# Comprehensive analysis of neoantigens derived from structural variation across whole genomes from 2528 tumors

**DOI:** 10.1186/s13059-023-03005-9

**Published:** 2023-07-17

**Authors:** Yang Shi, Biyang Jing, Ruibin Xi

**Affiliations:** 1 School of Mathematical Sciences, Peking University, Beijing, China; 2 School of Life Sciences, Peking University, Beijing, China; 3 Center for Statistical Science, Peking University, Beijing, China

**Keywords:** Neoantigen, Structural variation, Immunotherapy, Bioinformatics, Cancer vaccine, Tumor microenvironment

## Abstract

**Background:**

Neoantigens are critical for anti-tumor immunity and have been long-envisioned as promising therapeutic targets. However, current neoantigen analyses mostly focus on single nucleotide variations (SNVs) and indel mutations and seldom consider structural variations (SVs) that are also prevalent in cancer.

**Results:**

Here, we develop a computational method termed NeoSV, which incorporates SV annotation, protein fragmentation, and MHC binding prediction together, to predict SV-derived neoantigens. Analysis of 2528 whole genomes reveals that SVs significantly contribute to the neoantigen repertoire in both quantity and quality. Whereas most neoantigens are patient-specific, shared neoantigens are identified with high occurrence rates in breast, ovarian, and gastrointestinal cancers. We observe extensive immunoediting on SV-derived neoantigens, especially on clonal events, which suggests their immunogenic potential. We also demonstrate that genomic alteration-related neoantigen burden, which integrates SV-derived neoantigens, depicts the tumor-immune interplay better than tumor neoantigen burden and may improve patient selection for immunotherapy.

**Conclusions:**

Our study fills the gap in the current neoantigen repertoire and provides a valuable resource for cancer vaccine development.

**Supplementary Information:**

The online version contains supplementary material available at 10.1186/s13059-023-03005-9.

## Background

Somatic alterations in tumor genomes can generate mutated proteins, which, when broken down as peptide fragments and presented on major histocompatibility complex (MHC) molecules, can elicit anti-tumor immune responses [1]. These mutant peptides are commonly referred to as “neoantigens,” which comprise an important class of tumor antigens. T cells directed against neoantigens can drive the efficacy of immunotherapies [2–4]. The number of neoantigens has been demonstrated to be predictive of response to immune checkpoint blockade (ICB) across various cancer types [5]. Meanwhile, these tumor-specific neoantigens are neither subject to central immune tolerance nor likely to cause autoimmunity and thus are considered safe and promising therapeutic targets [6].

Recent years have seen major advances in next-generation sequencing (NGS). Its ability to identify somatic alterations in an ultrafast and effective way provides an unprecedented opportunity for developing neoantigen-targeting therapies. Adoptive transfer of autologous T cells that specifically targets somatic mutations has demonstrated effectiveness in multiple cancer types [7]. Neoantigen vaccines are shown to be able to generate neoantigen-specific T cells and induce tumor regression in melanoma and glioblastoma [8–10]. Moreover, recent clinical data indicates that in combination with PD-1 blockade, neoantigen-targeting therapies may generate a synergetic effect and produce broader antitumor responses, even in patients with “cold” tumor microenvironment [11, 12].

These neoantigen-based therapies start with the identification of the neoantigen repertoire for each patient. Previously, the best-studied mutation type is single nucleotide variants (SNV) on account of their high abundance in tumors and relative simplicity of detection [13]. However, since most SNVs merely alter a single amino acid in peptides, such neoantigens are likely to have a high degree of similarity to self-antigens, which may compromise MHC binding capability and the diversity of engaged T cell receptors (TCR) [14, 15]. In addition, therapies only targeting SNV-derived neoantigens cannot meet the medical needs of tumor entities with low SNV burdens [16]. Recent studies focusing on neoantigens created by other mutation types, such as short insertion and deletion (indel), intron retention, gene fusion, and alternative splicing, have shown the ability of these neoantigens to drive antitumor immunity and even mediate durable complete response in a fraction of patients [16–19]. Meanwhile, several accompanying computational tools have been developed to discover such neoantigens from NGS data, which dramatically enriched the neoantigen bank that could be targeted by immunotherapies [20–22].

Structural variation (SV), in which a genomic rearrangement of sizes ranging from single genes to whole chromosomes is amplified, deleted, or reordered, is another important class of alterations in cancer [23]. SV spreads widely in about 94.9% of tumors and thus constitutes a plentiful source for neoantigens, especially in cancer types with high SV loads such as sarcoma, esophagus cancer, and breast cancer [24]. Additionally, since SV often leads to novel open reading frame (ORF), it has the potential to generate neoantigens with lower self-similarity and higher immunogenicity. Recently, SV-derived neoantigens have been reported in mesothelioma [25] and demonstrated to be immunogenic in head and neck cancer [17]. However, there is still no comprehensive pan-cancer analysis of the neoantigenic potential of SV. In this study, using an in silico approach, we portrayed the landscape of SV-derived neoantigens from 2528 whole genomes across 30 cancer types and illustrated the paramount role of SV-derived neoantigens in understanding tumor-immune interactions and developing neoantigen-based therapies.

## Results

### Landscape of SV-derived neoantigens across cancer types

**Fig. 1:**
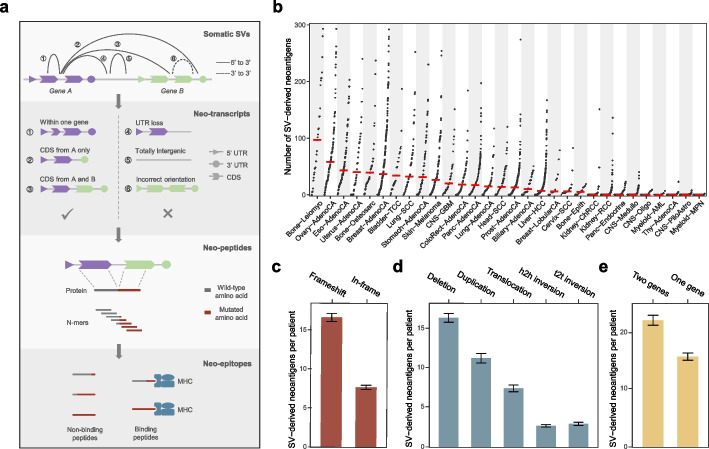
Prediction of SV-derived neoantigens across 2528 tumors. **a** Overview of the NeoSV workflow. **b** The number of SV-derived neoantigens per patient across 30 cancer types. Within each cancer type, patients are sorted by SV-derived neoantigen load. Red bars indicate median values. **c**–**e** The numbers of SV-derived neoantigens per patient categorized by the functional impact on proteins (**c**), SV type (**d**), and genomic location relative to genes (**e**). Medians ± s.e.m of the number of SV-derived neoantigens are plotted. h2h inversion, head-to-head inversion; t2t inversion, tail-to-tail inversion

We developed a computational pipeline named NeoSV to predict MHC I-restricted neoantigens from somatic SVs (Fig. 1a) (the “Methods” section). NeoSV first filters SVs with breakpoints at intergenic regions as well as SVs with “incorrect” orientations which could not generate functional transcripts. Next, NeoSV assembles the SV-derived neo-transcripts and in silico translated them to proteins. By applying a sliding window to the proteins, NeoSV obtains all possible short peptides. Only peptides with at least one mutated amino acid are retained to derive tumor-specific neo-peptides. At last, NeoSV predicts neo-peptides’ bindings to MHC molecules using NetMHCpan [26] and reports final candidate neoantigens.

Using this approach, we depicted the landscape of SV-derived neoantigens across 2528 tumor genomes from the Pan-Cancer Analysis of Whole Genomes (PCAWG) Consortium of the International Cancer Genome Consortium (ICGC) and The Cancer Genome Atlas (TCGA) [27] (Additional file 1: Table S1). The median numbers of SV-derived neoantigens varied considerably across different cancer types (0–97, only cancer types with > 10 tumors were included) (Fig. 1b and Additional file 2: Fig. S1). Cancer types with the largest numbers of SV-derived neoantigens were those bearing high levels of genomic instability, including ovary and breast adenocarcinoma characterized by DNA damage repair (DDR) deficiency [28], esophagus adenocarcinoma with frequent genomic catastrophes [29], and stomach adenocarcinoma with a subtype featured by high chromosomal instability (CIN) [30]. Bone leiomyoma and bone osteosarcoma were also observed with a high load of SV-derived neoantigens, which could be attributed to the high frequency of *TP53* and *RB1* mutations [31]. In contrast, hematological malignancies and brain tumors (except glioblastoma) rarely produced SV-derived neoantigens because of their relatively stable genomes (Fig. 1b) [32].

We further categorized the SV-derived neoantigens based on their genomic and functional characteristics. The majority of neoantigen-generating SVs had sizes ranging from 1 kb to 1 Mb (Additional file 2: Fig. S2a). As expected, frameshift SVs accounted for 82.4% neoantigens because they considerably altered the ORFs (Fig. 1c and Additional file 2: Fig. S2b). Nearly 68% of neoantigens were created by unbalanced genomic events like deletions and duplications owing to their high abundance in tumor genomes (Fig. 1d and Additional file 2: Fig. S2c) [24]. Notably, besides the SVs spanning two different genes, rearrangements in single genes, which were usually ignored by gene-fusion analyses, also accounted for 41.2% of the neoantigens (Fig. 1e and Additional file 2: Fig. S2d).

### SVs contribute to the neoantigen repertoire in terms of both quantity and quality

**Fig. 2:**
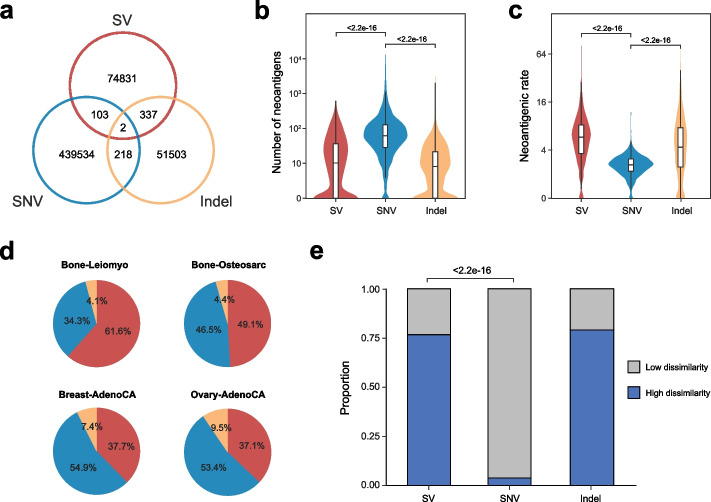
Comparison of the neoantigen repertoire derived from SVs to that from SNVs and indels. **a** Venn diagram of the intersection of neoantigen repertoire from SVs, SNVs, and indels. **b**, **c** Boxplots of the neoantigen load (**b**) and per-mutation neoantigenic rate (**c**) of every patient for SVs, SNVs, and indels (two-sided Wilcoxon rank-sum test). Boxplot hinges represent the 25th to 75th percentiles, and central lines represent median values; violin plots refer to the kernel probability densities. **d** Relative contributions of SVs, SNVs, and indels to the neoantigen repertoires of 4 representative cancer types. **e** Proportions of neoantigens with high or low self-similarity from SVs, SNVs, and indels (two-sided Fisher’s exact test)

In addition to SV, SNV and indel were the other two genomic sources of neoantigens. We compared neoantigens from different alteration types and found that the neoantigens generated by SV, SNV, and indel were almost mutually exclusive (Fig. 2a and Additional file 1: Table S2, S3). Overall, the number of SV-derived neoantigens per patient (median 9.0) was comparable to those derived from indels (median 7.0), though much fewer than SNV-derived neoantigens (median 65.0) (Fig. 2b). However, SV had a significantly higher neoantigenic rate (median 5.9) (the number of neoantigens generated per mutation) than SNV (median 1.8) and indel (median 4.0) (Fig. 2c) as a result of its damaging effect on ORFs. Remarkably, SV was the dominant source of neoantigens in bone leiomyoma (61.6%) and bone osteosarcoma (49.1%) and also contributed significantly to breast adenocarcinoma (37.7%) and ovary adenocarcinoma (37.1%) (Fig. 2d and Additional file 2: Fig. S3), which suggested its nonnegligible role in these cancer types.

Next, we compared several epitope-related metrics of neoantigens from SV, indel, and SNV. We found that binding affinity, binding stability, and hydrophobic fraction displayed no distinct differences among different mutation types (Additional file 2: Fig. S4). This was expected since these metrics were determined by the MHC-peptide interaction regardless of the genomic origin of neoantigens. To assess the self-dissimilarity, we searched the wild-type counterpart for each neoantigen throughout the peptidome using an in-house workflow (the “Methods” section). Our results showed that 76.5% of SV-derived neoantigens had no matched wild-type peptides, which was significantly higher than SNV-derived neoantigens (3.6%, *P* < 2.2e − 16) (Fig. 2e). Therefore, SV-derived neoantigens were more likely to engage T cells with diverse T cell receptors [15].

### SVs generate neoantigens shared across tumors

**Fig. 3:**
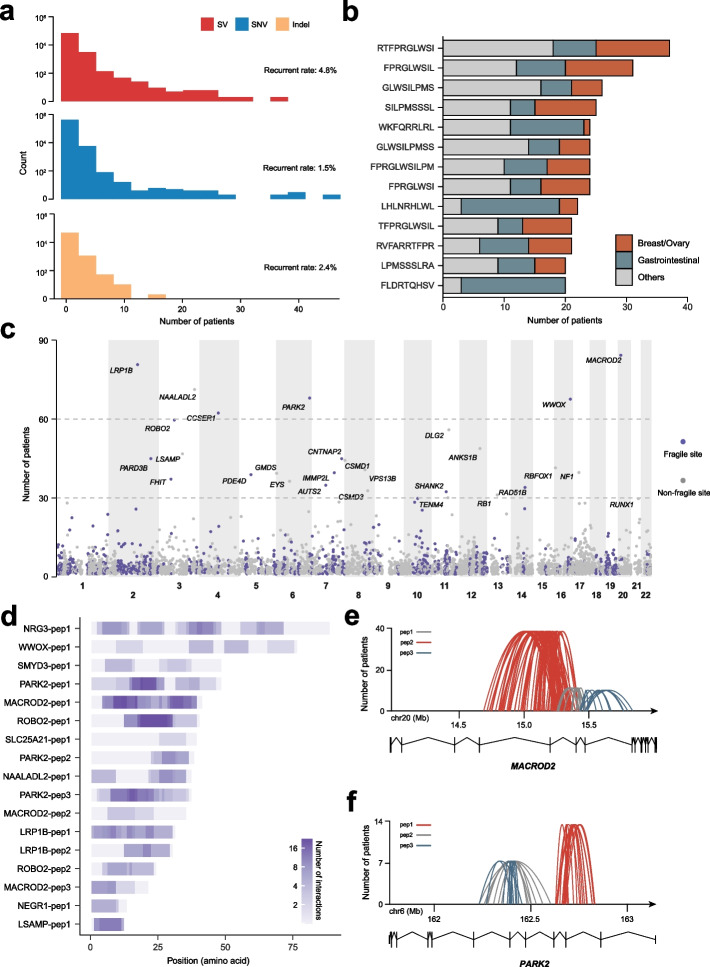
Shared SV-derived neoantigens. **a** Histograms showing the number of patients sharing a neoantigen created by SVs (top), SNVs (medium), and indels (bottom). Bin width: 5. **b** SV-derived neoantigens shared by at least 20 patients and their associated cancer types. **c** Genomic locations of the genes leading to shared SV-derived neoantigens. The *y*-axis represents the number of patients with SV-derived neoantigens. Different neoantigens originating from the same gene are summed. Genes are colored by overlap with fragile sites (purple) or not (grey). **d** Interactions between shared neo-peptides and 137 common MHC alleles. Suffixes of gene names are to discriminate the neo-peptides from the same gene. The *x*-axis indicates the position relative to the first mutated amino acid of the neo-peptide. The color gradient indicates the number of MHC alleles that can bind to each *k*-mer (log-transformed). Only neo-peptides shared by > 5 patients and have > 1 interactions are listed. **e**, **f** Breakpoint junctions of the SVs leading to neo-peptides of *MACROD2* (**e**) and *PARK2* (**f**). Each arc represents an SV and is colored according to the identity of the neo-peptide. The exon (vertical line)-intron (horizontal broken line) structures of genes are displayed at the bottom

Vaccines targeting the neoantigens shared across patients are constantly pursued for their cost efficiency and easy developmental routes. Several recurrent SNVs and indels were reported to create shared neoantigens that induced anti-tumor immunity [33]. To investigate shared neoantigens from SVs, we calculated the occurrence of each SV-derived neoantigen in PCAWG (Fig. 3a). As expected, the majority of SV-derived neoantigens were patient-specific, and only a handful of them occurred recurrently (> 2 patients). However, the frequency of recurrent SV-derived neoantigens (4.8%) was higher than those from SNVs (1.5%) and indels (2.4%). When focused on the neoantigens shared by at least 20 patients, we found them enriched in breast, ovarian, and gastrointestinal cancers (including esophagus, stomach, and colorectal adenocarcinoma) (Fig. 3b). For example, FLDRTQHSV was rare in the pan-cancer cohort (0.7%), but had a frequency as high as 8% in gastrointestinal cancers (Additional file 2: Fig. S5), thus was attractive for developing cancer vaccines. Additionally, we mapped the SV-derived neoantigens to genes and found most genes giving rise to shared neoantigens were located on chromosomal fragile sites (Fig. 3c) [34], such as *LRP1B*, *MACROD2*, *WWOX*, and *PARK2*. The oncogenic role of these genes remained controversial [35]. However, their peptidome had the potential to be targeted by immunotherapies.

We noted that some SV-derived neo-peptides (the mutated part of an SV-disrupted protein) were shared among patients, but could not be presented by MHCs, thus were not identified as shared neoantigens in PCAWG. However, these neo-peptides still have the value as therapeutic targets if they could bind to other MHC alleles. Therefore, we collected 135 globally most prevalent MHC alleles and studied their interactions with these shared neo-peptides [36]. For every neo-peptide (occurrence in > 5 patients), we in silico generated all possible *k*-mers (8–11) and predicted their binding affinities with the common MHC alleles (Additional file 1: Table S4). Extensive interactions (binding between one *k*-mer and one MHC allele) emerged alongside the shared neo-peptides (Fig. 3d and Additional file 1: Table S5). For example, the recurrent deletion of exon5 in *MACROD2* and exon9 in *PARK2* had substantial interactions with the common MHC alleles (Fig. 3e, f). When stratified by cancer type, we found these shared neo-peptides were also enriched in ovarian, breast, and gastrointestinal cancers (Additional file 2: Fig. S5). Taken together, our data demonstrated the presence of shared SV-derived neoantigens and suggested the possibility of developing off-the-shelf vaccines for specific malignancies.

### Negative selection from immune surveillance on SV-derived neoantigens

**Fig. 4:**
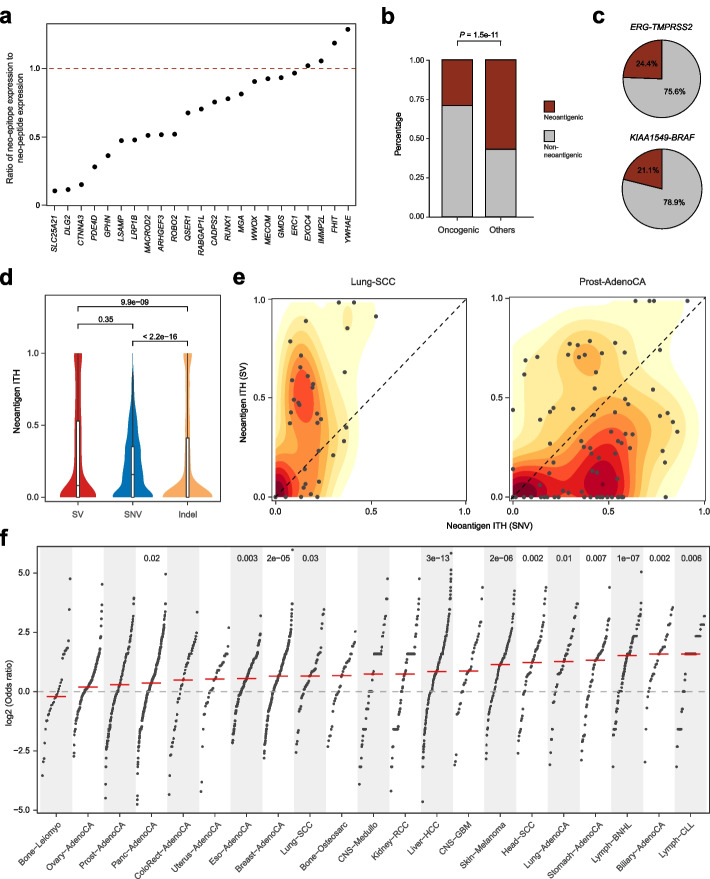
Negative selection pressure on SV-derived neoantigens. **a** Given a shared neo-peptide from a gene, samples that bear the neo-peptide are retrieved and grouped to neoantigen and neo-peptide samples according to whether they could present the neo-peptide or not. The ratio between the average expressions of the gene in the neoantigen and neo-peptide samples is shown. **b** Proportions of neoantigenic SVs in oncogenic SVs and passenger SVs (two-sided Fisher’s exact test). **c** Proportions of *TMPRSS2*-*ERG* and *KIAA1549*-*BRAF* fusions that result in neoantigens and do not result in neoantigens. **d** Comparison of patient-level ITH of SV-, SNV-, and indel-derived neoantigens (two-sided Wilcoxon rank-sum test). Boxplot hinges represent 25th to 75th percentiles, and central lines represent median values; violin plots refer to the kernel probability densities. **e** 2D density plots showing the ITH of SV-derived neoantigens versus that of SNV-derived neoantigens in lung squamous carcinoma (Lung-SCC, left) and prostate adenocarcinoma (Prost-AdenoCA, right). **f** Distributions of odds ratios of generating neoantigens from subclonal and clonal SVs in different patients. The odds ratios are log-transformed. Red bars indicate median values. Cancer types with median odds ratio significantly deviated from 1 are labeled (one-sample Wilcoxon rank-sum test)

Neoantigens can be selectively lost from tumor cells by reduced gene expression [37, 38]. However, since frameshift SVs might lead to premature stop codons, nonsense-mediated mRNA decay (NMD) could also result in a decrease in the expression of genes affected by SVs [39]. To avoid the confounding effect of NMD, for each gene, we restricted our analysis to tumors with the same neo-peptides, thus bearing similar degrees of NMD (Additional file 2: Fig. S6a). Among the genes with enough data (> 2 tumors) for comparison, we observed that genes which could be presented as neoantigens had moderately lower expressions compared to those only generating neo-peptides but could not be presented by MHC (Fig. 4a). To avoid the impact of cancer type on gene expression, we further normalized the expression values to *Z*-scores within each cancer type and found consistent results (Additional file 2: Fig. S6b). It indicated that SV-derived neoantigens probably were subject to modest expression reduction as a result of the negative selection pressure from immune cells.

Recent data showed that oncogenic point mutations were biased toward peptides that are poorly presented by MHC [40]. We hypothesized that such bias might also apply to SVs. Based on the annotation of gene fusions from The Cancer Gene Census [41], we categorized SVs into oncogenic ones and passengers. We found that in contrast to passengers, oncogenic SVs were less likely to generate neoantigens (56.9% vs 29.1%, odds ratio = 0.31, *P* = 1.5e − 11) (Fig. 4b). Such depletion effect was still significant (*P* = 3.79e − 8) after controlling the number of affected amino acids and the frameshift effect in a logistic model (Additional file 1: Table S6 and the “Methods” section). For example, only 21.1% of the recurrent *KIAA1549*-*BRAF* fusion in pilocytic astrocytoma and 24.4% of *ERG*-*TMPRSS2* in prostate adenocarcinoma led to neoantigens (Fig. 4c). These data suggested that oncogenic SVs were restricted by immune surveillance and tended to be poorly presented.

### SV-derived neoantigens throughout tumor evolution

Neoantigen intratumor heterogeneity (ITH) could influence antitumor immunity and response to ICB [42]. We used the fraction of subclonal SV-derived neoantigens to estimate the neoantigen ITH (Additional file 2: Fig. S7 and Additional file 1: Table S7). The median ITH of SV-derived neoantigens was comparable to those from SNVs (*P* = 0.35) but significantly higher than indels (*P* < 2.2e − 16) (Fig. 4d). Nevertheless, when we did patient-by-patient comparisons, the ITH of SV-derived neoantigens showed different patterns from SNV-derived neoantigens (Additional file 2: Fig. S8). For example, 76% of lung squamous cell carcinomas had greater ITH of SV-derived neoantigens, whereas 60% of prostate adenocarcinoma displayed higher ITH of SNV-derived neoantigens (Fig. 4e). It suggested that different types of neoantigens might emerge at different stages of tumor progression and bear different selective pressures from the immune system.

To investigate the change of immunoediting effect on SV-derived neoantigens during tumor evolution, we calculated the odds ratio (OR) of generating neoantigen from clonal and subclonal SVs for each tumor. We found that neoantigen-generating SVs were enriched in subclonal SVs (OR > 1) in the majority of cancer types (Fig. 4f). Interestingly, such enrichment was statistically significant in hematological malignancies and some well-known “hot” solid tumors, such as lung squamous cell carcinoma (*P* = 0.03), lung adenocarcinoma (*P* = 0.01), head squamous cell carcinoma (*P* = 0.002), liver hepatocellular carcinoma (*P* = 3e − 13) and melanoma (*P* = 2e − 6) (Fig. 4f). Indeed, the immune infiltration of these cancer types were also among the top ones in the PCAWG cohort (Additional file 2: Fig. S9), and thus might provide stronger anti-tumor immunity to eliminate neoantigenic tumor cells at the stage of tumor initiation.

### GANB characterizes the immunogenomic features of tumor more comprehensively than TNB

**Fig. 5:**
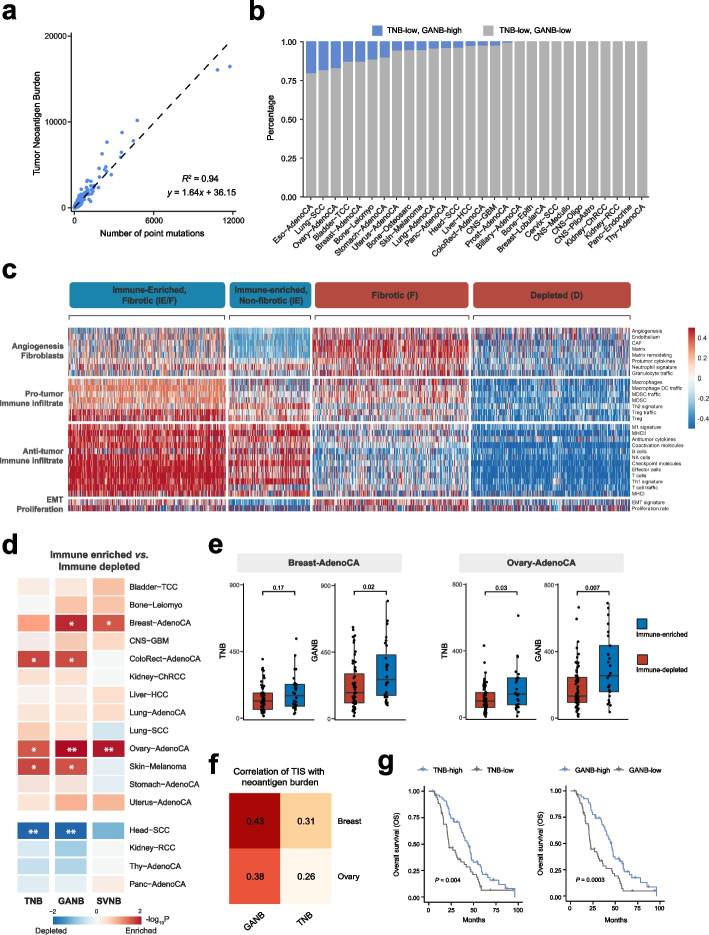
GANB captures the immunogenomic characteristics of tumor cells. **a** Relationship between tumor neoantigen burden (*y*-axis) and tumor mutation burden (*x*-axis). The linear regression line is shown in the plot. **b** Proportions of TNB-low patients that can be re-defined by GANB as GANB-high across cancer types. **c** Heatmap of 1188 PCAWG tumors (columns) classified into four distinct TME subtypes based on unsupervised clustering of the 29 pre-defined gene signatures (rows). **d** Heatmap of the differences in TNB (left), GANB (middle), and SVNB (right) between immune-enriched tumors and immune-depleted tumors (two-sided Wilcoxon rank-sum test). Red indicates a higher neoantigen load in immune-enriched tumors while blue indicates a higher neoantigen load in immune-depleted tumors. *P < 0.05, **P < 0.005. **e** Differences in TNB/GANB between immune-enriched and immune-depleted tumors from Breast-AdenoCA (left) and Ovary-AdenoCA (right) (two-sided Wilcoxon rank-sum test). **f** Correlation between TIS and neoantigen load measured by TNB or GANB in Breast-AdenoCA and Ovary-AdenoCA (Spearman correlation). **g** Kaplan–Meier curves of patients with ovary adenocarcinoma stratified by TNB (left) and GANB (right), using median values as cutoffs (two-sided rank sum test)

Recently, the United States Food and Drug Administration (FDA) approved pembrolizumab (anti-PD-1) for the treatment of unresectable and metastatic tumors with high tumor mutation burden (TMB-High). However, a fraction of patients judged as TMB-Low also respond to pembrolizumab [43], which may be explained by the fact that TMB and its derivative tumor neoantigen burden (TNB) did not consider SV-derived neoantigen burden (SVNB). Therefore, we proposed genomic alteration-related neoantigen burden (GANB), which integrated neoantigens from SNVs, indels, and SVs together, to fully capture the immunogenomic characteristics of tumors (Additional file 1: Table S8). First, we investigated whether GANB could refine patient selection for anti-PD-1 therapy. According to the linear relationship between TNB and TMB, we transformed the threshold of TMB-High (> 175 mutations per exome) to a neoantigen-based threshold: TNB-High (> 323 neoantigens per exome) (Fig. 5a) [44]. Using this criterion, we found a significant proportion of TNB-low patients, such as 20.6% esophagus adenocarcinomas, 18.7% lung squamous cell carcinoma, 17.4% ovary adenocarcinoma, and 13.2% breast adenocarcinoma, had sufficient neoantigens (> 323) if assessed by GANB (Fig. 5b), thus potentially could benefit from anti-PD-1 therapy and should not be excluded from the pembrolizumab treatment.

Next, we investigated the association of GANB with tumor microenvironment (TME). Following a previously established TME subtyping framework [45], we clustered the TME of 1188 transcriptomes from PCAWG into four subtypes: (1) immune-enriched, fibrotic (IE/F); (2) immune-enriched, non-fibrotic (IE); (3) fibrotic (F); and (4) immune-depleted (D) (Fig. 5c, Additional file 2: Fig. S9 and Additional file 1: Table S9). In most cancers, a higher neoantigen load was observed in immune-enriched tumors (IE and IE/F) than immune-depleted tumors (D and F) (Fig. 5d), which could be attributed to the anti-tumor immunity elicited by neoantigens. Notably, for breast and ovary adenocarcinoma, SVNB was significantly higher in immune-enriched tumors, thus making GANB better correlated with immune infiltration than TNB (Fig. 5e). To validate such phenomenon, we used another widely used biomarker, tumor inflammation signature (TIS), to reflect the level of tumor infiltrated lymphocytes (TILs) [46]. Similarly, stronger positive correlations with TIS were observed in GANB than TNB (Fig. 5f). The mutation load has been reported as a prognosis biomarker for ovarian cancer [47], and we further checked if considering SV-derived neoantigens could improve the prognostic value. Indeed, our analysis showed that GANB displayed better patient stratification than TNB (Fig. 5g and Additional file 2: Fig. S10).

## Discussion

In this study, we developed a computational workflow to predict neoantigens from SVs and depicted the landscape of SV-derived neoantigens from 2528 whole genomes. We demonstrated SV as an important source of neoantigens, considering their 15% overall abundance (quantity) and higher self-dissimilarity than SNVs (quality). Additionally, we comprehensively analyzed the occurrence and expression of SV-derived neoantigens in relation to TME, oncogenic role, and clonal evolution, unraveling their extensive participation in immune surveillance and tumor evasion. Furthermore, we provided a list of shared SV-derived neoantigens as putative targets of cancer vaccines. Our analysis highlighted the important role of this novel source of neoantigens in driving antitumor immunity and developing neoantigen-based immunotherapies.

Although SNVs contributed greatly (77%) to the neoantigen repertoire of the PCAWG population, SVs also made a significant contribution relative to their low number. In tumors with low mutation burdens, SVs might be of greater importance, as illustrated by the fact that SVs accounted for > 50% of the neoantigens in sarcoma. Meanwhile, these SV-derived neoantigens were attractive immunotherapy targets in fusion-driven cancers. Though these recurrent SVs were biased toward generating poorly presented peptides, their overall high occurrence frequencies mitigated such depletion effect. Besides, oncogenic SVs were usually the driving force of cell proliferation, and thus reduction of their expressions would be disadvantageous for tumor cells.

In addition to the well-studied driver fusions, we discovered genes located on fragile sites, such as *MACROD2*, *ROBO2*, and *FHIT*, also produced shared neoantigens. Although the exact role of these genes in tumor biology remained controversial, their high occurrence frequencies in tumor patients made them ideal targets for cancer vaccines. In addition, our result showed that the shared neo-peptides created by these SVs were not only presented by MHCs in the PCAWG population but could also form extensive bindings to other globally common MHC alleles, and thus more patients with different genetic backgrounds might benefit.

In accordance with previously reported immunoediting effect on SNV-derived neoantigens [48], we observed negative selection on SV-derived neoantigens by the immune system, especially on clonal events, which might be due to the higher immunogenicity of clonal neoantigens. However, as current computational methods for assessing immunogenicity could be inaccurate [49], further experiments were required to validate this hypothesis. Meanwhile, late-stage tumors can escape from immune surveillance via immune exclusion or inducing T cell exhaustion [50]. Thus, it is possible that the change in external microenvironment during tumor evolution, instead of the internal properties of neoantigens, led to clonal neoantigen depletion. Nevertheless, clonal neoantigens were still more important therapy targets than subclonal ones in that targeting them could potentially kill more tumor cells.

During the review process, we noted that Neoantimon [51] can also predict neoantigens from SVs. We compared the SV-derived neoantigens predicted by Neoantimon and our tool NeoSV and found a high degree of consistency: 94% of the predictions were common. There are some minor differences between the two algorithms. First, Neoantimon ignores read-through SVs (missing the stop codon and being translated to the poly-A tail), while NeoSV includes them with a specific “read-through” label. Second, Neoantimon relies on the UCSC database, while NeoSV depends on the Ensembl database, which occasionally leads to neoantigen prediction differences. Third, Neoantimon requires pre-annotated SVs, while NeoSV is a one-stop solution which accepts raw VCF (variant call format) file and thus is more convenient for users. INTEGRATE-neo is another tool that can predict neoantigens from gene fusions [52]. However, as shown in Fig. 1e, nearly half of the SV-derived neoantigens were located within one gene and thus could not be covered by fusion-centric neoantigen prediction tools. In addition, these SVs usually spanned more than 5 kb and were often ignored by indel detection algorithms. Thus, neoantigens from these SVs would also be missed by indel-centric neoantigen analysis [16].

Our analysis has several limitations. First, a recent study pointed out that noncoding regions can be a major source of neoantigens [53], suggesting the importance of intergenic SVs in neoantigen prediction. However, because of the computational challenge of gene boundary prediction, we did not consider SVs in intergenic regions. Future improvements on “top-down” strategies like MHC-binding peptide mass spectrometry could help to better study these uncanonical neoantigens [54]. Second, we chose the isoform with the longest coding sequence (CDS) for neoantigen analysis, but this isoform might not be the one used in tumor cells. Although 46% of PCAWG samples have matched RNA-seq data, inferring the used isoform from short reads remained a challenge [55]. The third generation sequencing, such as single-molecule real-time sequencing [56], can profile full-length isoforms and thus could help to improve the prediction of SV-derived neoantigens. Third, our analysis relied on the in silico MHC binding prediction. The conclusions in the paper were based on the NetMHCpan and could also be repeated using another well-known MHC binding prediction algorithm MHCflurry [57] (Additional file 2: Fig. S11). However, considering the discrepancies between in silico predictions and experimental results [49], whether these predicted neoantigens could elicit T cell responses need further experimental validation.

Recently, a phase 2 trial targeting personalized neoantigens (KEYNOTE-082) in melanoma has met its primary efficacy endpoint. Meanwhile, several neoantigen-based trials for other cancers such as colorectal, pancreatic, and lung cancers are ongoing [58]. It is anticipated that the combination of immune checkpoint inhibitors with neoantigen vaccines would take cancer treatment into a new era. However, until now, all vaccines were designed only for neoantigens from SNVs and indels, which resulted in tremendous unmet medical needs for patients with few canonical point mutations. Therefore, fully taking advantage of other types of cancer alterations, such as SVs and noncoding variants will be important for future drug development.

## Conclusion

Our comprehensive analysis of 2528 whole genomes unveiled the immunogenic properties of SVs, a never-touched neoantigen source. We demonstrated that SV-derived neoantigens were of paramount value, in both quantity and quality, for developing cancer vaccines. We also provided compelling evidence that SV-derived neoantigens bridged the tumor-immune interaction, thus were important for future immune-oncology studies.

## Methods

### Neoantigen prediction from SVs

We developed NeoSV for neoantigen prediction from SVs. SVs in VCF format with the genomic location of breakpoints and junction orientations were required as input. First, all breakpoints were mapped to an annotated transcript database (Ensembl v75) and SVs with intergenic breakpoints were removed. For genes with multiple transcripts, the one with the longest coding region was chosen for analysis. Then, the “neo-transcripts” were assembled in 5′ to 3′ orientation. SVs which could not generate transcripts with intact 5′UTR-CDS-3′UTR structures were discarded during this process.

Next, the “neo-transcripts” were translated to “neo-proteins” according to the standard codon table. For SVs with a start codon loss, a downstream start codon could be automatically detected and used as a new translational starting site. However, these genes were not included in this study due to the uncertainty of predicting translation starting sites. For all frameshift SVs, translation was terminated until the first stop codon or the 3-prime boundary of the transcript was reached.

These “neo-proteins” were then fragmented into short peptides (8–11 residues) using sliding windows. By comparing with wild-type proteins, only peptides with at least one non-self-residue were retained to get tumor-specific short peptides. The binding probabilities of peptides to MHC molecules were then predicted using NetMHCpan [26]. Finally, peptides with IC_50_ < 500 nM and rank < 2.0 were selected as “neoantigens” for analysis in this study.

### Neoantigen prediction from SNVs and indels

SNVs and indels were annotated using Oncotator [59]. Non-silent mutations were further included for neoantigen prediction using Topiary (https://github.com/openvax/topiary) with the threshold of IC_50_ set to 500 nM and rank set to 2.0. No filtering on gene expression was applied.

### Self-dissimilarity of neoantigens

All annotated genes in Ensembl were in silico translated and cleaved into 8–11 mer peptides to get the peptidome of normal cells. Then, each peptide was compared throughout the peptidome using blast to find the most similar counterpart [60]. A neoantigen was defined as “high-similarity” if it had a counterpart with an alignment score > 35.

### Hydrophobicity and binding stability of neoantigens

Hydrophobicity fraction was calculated as the fraction of amino acids that were hydrophobic, namely V, I, L, F, M, W, and C. The binding stability of neoantigens to MHC molecules was calculated by NetMHCStabPan with default parameters [61].

### TME analysis

Gene set variation analysis (GSVA) was used to calculate the scores of 29 TME-related signatures in each tumor [62]. Then *K*-means clustering was applied to cluster tumors into four subtypes using Euclidean distances. These subtypes were annotated according to previously described consensus clusters in TCGA.

### Statistical analysis

When assessing the neoantigen depletion in oncogenic SVs, a logistic regression model was used to control the confounding variables:

$$\mathrm{logit}\left(P\right)={\beta }_{0}+{\beta }_{1}{X}_{1}+{\beta }_{2}{X}_{2}+{\beta }_{3}{X}_{3},$$

where $P$ represented the probability of an SV generating a neoantigen, ${X}_{1}$ represented whether this SV was oncogenic, ${X}_{2}$ was the number of affected amino acids, and ${X}_{3}$ represented whether it was a frameshift SV.

The chi-squared test was used for the assessment of the enrichment of binary features. Odds ratios were calculated with the Haldane-Anscombe correction to avoid division by zero. The correlation between two continuous variables was assessed by Spearman correlation. Differences in the medians of continuous variables between the two groups were assessed by the Wilcoxon rank-sum test. Median survivals were estimated using the Kaplan–Meier method. Log-rank test was used to compare survival curves between subgroups. The significance level for all comparisons was 0.05 unless indicated otherwise. All statistical analyses and visualizations were performed with R (v.4.0.2).

## Supplementary Information

**Additional file 1: Supplementary figures** **(Fig. S1 to Fig. S11).**

**Additional file 2: Supplementary Table S1 to Table S9.** It contains the genomic position, peptide sequence, and binding affinity of all predicted neoantigens; the list of recurrent SV-neoantigens and their binding properties with high-prevalence HLA alleles; the intratumor heterogeneity of neoantigen per patient; the neoantigen burden (TNB and GANB) per patient; the tumor microenvironment subtype of each patient.

**Additional file 3. **Review history.

## Data Availability

SNV, indel, SV, MSI, HLA genotype, clonality, and gene expression data were retrieved from the official release of the PCAWG working group [27]. The ICGC portion was accessed via the ICGC data portal (https://dcc.icgc.org/releases/PCAWG/) under a DACO-authorized account. The TCGA portion was accessed through dbGAP (phs000178) [63, 64]. HLA types inferred by ALPHLARD were retrieved from a previous work [65]. The processed data for this presented analysis is in the supplementary tables. The code for reproducing the presented analysis results is available at Zenodo [66]. NeoSV is available under the MIT license at GitHub [67]. No other scripts, software, and data were used other than those mentioned in the “Methods” section.

## References

[1] Schumacher TN, Schreiber RD (2015). Neoantigens in cancer immunotherapy. Science.

[2] Gubin MM (2014). Checkpoint blockade cancer immunotherapy targets tumour-specific mutant antigens. Nature.

[3] Chan TA, Wolchok JD, Snyder A (2015). Genetic basis for clinical response to CTLA-4 Blockade in melanoma. N Engl J Med.

[4] Rizvi, N. A. et al. Cancer immunology. Mutational landscape determines sensitivity to PD-1 blockade in non-small cell lung cancer. Science 348, 124–128, 10.1126/science.aaa1348 (2015). 10.1126/science.aaa1348 PMC4993154 25765070

[5] Samstein RM (2019). Tumor mutational load predicts survival after immunotherapy across multiple cancer types. Nat Genet.

[6] Yarchoan M, Johnson BA, Lutz ER, Laheru DA, Jaffee EM (2017). Targeting neoantigens to augment antitumour immunity. Nat Rev Cancer.

[7] Morotti M (2021). Promises and challenges of adoptive T-cell therapies for solid tumours. Br J Cancer.

[8] Sahin U (2017). Personalized RNA mutanome vaccines mobilize poly-specific therapeutic immunity against cancer. Nature.

[9] Keskin DB (2019). Neoantigen vaccine generates intratumoral T cell responses in phase Ib glioblastoma trial. Nature.

[10] Hilf N (2019). Actively personalized vaccination trial for newly diagnosed glioblastoma. Nature.

[11] Martin-Broto, J. et al. Pazopanib for treatment of typical solitary fibrous tumours: a multicentre, single-arm, phase 2 trial. Lancet Oncol 21, 456–466,10.1016/S1470-2045(19)30826-5 (2020). 10.1016/S1470-2045(19)30826-5 32066540

[12] Ott PA (2017). An immunogenic personal neoantigen vaccine for patients with melanoma. Nature.

[13] Smith CC (2019). Alternative tumour-specific antigens. Nat Rev Cancer.

[14] Ghorani E (2018). Differential binding affinity of mutated peptides for MHC class I is a predictor of survival in advanced lung cancer and melanoma. Ann Oncol.

[15] Nelson RW (2015). T cell receptor cross-reactivity between similar foreign and self peptides influences naive cell population size and autoimmunity. Immunity.

[16] Turajlic S (2017). Insertion-and-deletion-derived tumour-specific neoantigens and the immunogenic phenotype: a pan-cancer analysis. Lancet Oncol.

[17] Yang W (2019). Immunogenic neoantigens derived from gene fusions stimulate T cell responses. Nat Med.

[18] Kahles, A. et al. Comprehensive analysis of alternative splicing across tumors from 8,705 patients. Cancer Cell 34, 211–224 e216, 10.1016/j.ccell.2018.07.001 (2018). 10.1016/j.ccell.2018.07.001 PMC9844097 30078747

[19] Smart AC (2018). Intron retention is a source of neoepitopes in cancer. Nat Biotechnol.

[20] Fotakis G, Rieder D, Haider M, Trajanoski Z, Finotello F (2020). NeoFuse: predicting fusion neoantigens from RNA sequencing data. Bioinformatics.

[21] Hundal J (2016). pVAC-Seq: A genome-guided in silico approach to identifying tumor neoantigens. Genome Med.

[22] Chai, S. et al. NeoSplice: a bioinformatics method for prediction of splice variant neoantigens. Bioinform Adv 2, vbac032, 10.1093/bioadv/vbac032 (2022). 10.1093/bioadv/vbac032 PMC9154024 35669345

[23] Ho SS, Urban AE, Mills RE (2020). Structural variation in the sequencing era. Nat Rev Genet.

[24] Li Y (2020). Patterns of somatic structural variation in human cancer genomes. Nature.

[25] Mansfield, A. S., Peikert, T. & Vasmatzis, G. Chromosomal rearrangements and their neoantigenic potential in mesothelioma. Transl Lung Cancer Res 9, S92-S99, 10.21037/tlcr.2019.11.12 (2020). 10.21037/tlcr.2019.11.12 PMC7082253 32206575

[26] Jurtz, V. et al. NetMHCpan-4.0: improved peptide-MHC class I interaction predictions integrating eluted ligand and peptide binding affinity data. J Immunol 199, 3360–3368, 10.4049/jimmunol.1700893 (2017). 10.4049/jimmunol.1700893 PMC5679736 28978689

[27] Consortium, I. T. P.-C. A. o. W. G. Pan-cancer analysis of whole genomes. Nature 578, 82–93, 10.1038/s41586-020-1969-6 (2020). 10.1038/s41586-020-1969-6 PMC7025898 32025007

[28] O’Connor MJ (2015). Targeting the DNA damage response in cancer. Mol Cell.

[29] Nones K (2014). Genomic catastrophes frequently arise in esophageal adenocarcinoma and drive tumorigenesis. Nat Commun.

[30] Cancer Genome Atlas Research, N. Comprehensive molecular characterization of gastric adenocarcinoma. Nature 513, 202–209, 10.1038/nature13480 (2014). 10.1038/nature13480 PMC4170219 25079317

[31] Bousquet M (2016). Whole-exome sequencing in osteosarcoma reveals important heterogeneity of genetic alterations. Ann Oncol.

[32] Taylor, A. M. et al. Genomic and functional approaches to understanding cancer aneuploidy. Cancer Cell 33, 676–689 e673, 10.1016/j.ccell.2018.03.007 (2018). 10.1016/j.ccell.2018.03.007 PMC6028190 29622463

[33] Lang F, Schrors B, Lower M, Tureci O, Sahin U (2022). Identification of neoantigens for individualized therapeutic cancer vaccines. Nat Rev Drug Discov.

[34] Kumar R (2019). HumCFS: a database of fragile sites in human chromosomes. BMC Genomics.

[35] Glover TW, Wilson TE, Arlt MF (2017). Fragile sites in cancer: more than meets the eye. Nat Rev Cancer.

[36] Gonzalez-Galarza FF (2020). Allele frequency net database (AFND) 2020 update: gold-standard data classification, open access genotype data and new query tools. Nucleic Acids Res.

[37] Verdegaal EM (2016). Neoantigen landscape dynamics during human melanoma-T cell interactions. Nature.

[38] Balachandran VP (2017). Identification of unique neoantigen qualities in long-term survivors of pancreatic cancer. Nature.

[39] Tan K, Stupack DG, Wilkinson MF (2022). Nonsense-mediated RNA decay: an emerging modulator of malignancy. Nat Rev Cancer.

[40] Marty, R. et al. MHC-I genotype restricts the oncogenic mutational landscape. Cell 171, 1272–1283 e1215, 10.1016/j.cell.2017.09.050 (2017). 10.1016/j.cell.2017.09.050 PMC5711564 29107334

[41] Tate JG (2019). COSMIC: the Catalogue Of Somatic Mutations In Cancer. Nucleic Acids Res.

[42] McGranahan N (2016). Clonal neoantigens elicit T cell immunoreactivity and sensitivity to immune checkpoint blockade. Science.

[43] McGrail DJ (2021). High tumor mutation burden fails to predict immune checkpoint blockade response across all cancer types. Ann Oncol.

[44] Cristescu, R. et al. Tumor mutational burden predicts the efficacy of pembrolizumab monotherapy: a pan-tumor retrospective analysis of participants with advanced solid tumors. J Immunother Cancer 10, 10.1136/jitc-2021-003091 (2022). 10.1136/jitc-2021-003091 PMC8804694 35101941

[45] Bagaev, A. et al. Conserved pan-cancer microenvironment subtypes predict response to immunotherapy. Cancer Cell 39, 845–865 e847, 10.1016/j.ccell.2021.04.014 (2021). 10.1016/j.ccell.2021.04.014 34019806

[46] Cristescu, R. et al. Pan-tumor genomic biomarkers for PD-1 checkpoint blockade-based immunotherapy. Science 362, 10.1126/science.aar3593 (2018). 10.1126/science.aar3593 PMC6718162 30309915

[47] Shen S (2019). Development and validation of an immune gene-set based prognostic signature in ovarian cancer. EBioMedicine.

[48] Rooney MS, Shukla SA, Wu CJ, Getz G, Hacohen N (2015). Molecular and genetic properties of tumors associated with local immune cytolytic activity. Cell.

[49] Wells, D. K. et al. Key parameters of tumor epitope immunogenicity revealed through a consortium approach improve neoantigen prediction. Cell 183, 818–834 e813, 10.1016/j.cell.2020.09.015 (2020). 10.1016/j.cell.2020.09.015 PMC7652061 33038342

[50] Joyce JA, Fearon DT (2015). T cell exclusion, immune privilege, and the tumor microenvironment. Science.

[51] Hasegawa T (2020). Neoantimon: a multifunctional R package for identification of tumor-specific neoantigens. Bioinformatics.

[52] Zhang J, Mardis ER, Maher CA (2017). INTEGRATE-neo: a pipeline for personalized gene fusion neoantigen discovery. Bioinformatics.

[53] Laumont, C. M. et al. Noncoding regions are the main source of targetable tumor-specific antigens. Sci Transl Med 10, 10.1126/scitranslmed.aau5516 (2018). 10.1126/scitranslmed.aau5516 30518613

[54] Abelin JG (2017). Mass spectrometry profiling of HLA-associated peptidomes in mono-allelic cells enables more accurate epitope prediction. Immunity.

[55] Steijger T (2013). Assessment of transcript reconstruction methods for RNA-seq. Nat Methods.

[56] Ardui S, Ameur A, Vermeesch JR, Hestand MS (2018). Single molecule real-time (SMRT) sequencing comes of age: applications and utilities for medical diagnostics. Nucleic Acids Res.

[57] O’Donnell, T. J., Rubinsteyn, A. & Laserson, U. MHCflurry 2.0: improved pan-allele prediction of MHC class I-presented peptides by incorporating antigen processing. Cell Syst 11, 418–419, 10.1016/j.cels.2020.09.001 (2020). 10.1016/j.cels.2020.09.001 33091335

[58] Lin MJ (2022). Cancer vaccines: the next immunotherapy frontier. Nat Cancer.

[59] Ramos AH (2015). Oncotator: cancer variant annotation tool. Hum Mutat.

[60] Altschul SF, Gish W, Miller W, Myers EW, Lipman DJ (1990). Basic local alignment search tool. J Mol Biol.

[61] Rasmussen M (2016). Pan-specific prediction of peptide-MHC class I complex stability, a correlate of T Cell immunogenicity. J Immunol.

[62] Hanzelmann S, Castelo R, Guinney J (2013). GSVA: gene set variation analysis for microarray and RNA-seq data. BMC Bioinformatics.

[63] Cancer Genome Atlas Research, N. et al. The Cancer Genome Atlas Pan-Cancer analysis project. Nat Genet 45, 1113–1120, 10.1038/ng.2764 (2013). 10.1038/ng.2764 PMC3919969 24071849

[64] National Institutes of Health. The Cancer Genome Atlas (TCGA). Database of Genotypes and Phenotypes (dbGaP). phs000178. (2019).

[65] Yang, F. et al. Quantifying immune-based counterselection of somatic mutations. PLoS Genet 15, e1008227, 10.1371/journal.pgen.1008227 (2019). 10.1371/journal.pgen.1008227 PMC6657826 31344031

[66] Shi, Y., Jing, B. & Xi. R. Pan-cancer analysis of SV-derived neoantigens. Zenodo 10.5281/zenodo.8060411 (2023).

[67] Shi, Y. A computational workflow to identify neoantigens from structural variations. Github https://github.com/ysbioinfo/NeoSV (2023).

