# Supplementary material for: Comprehensive analysis of neoantigens derived from structural variation across whole genomes from 2528 tumors
Source: Genome Biol. 2023 Jul 17;24:169. doi: 10.1186/s13059-023-03005-9 (PMC10351168; doi:10.1186/s13059-023-03005-9)
Supplement: Supplementary file 2 — Additional file 2: Supplementary Table S1 to Table S9. It contains the genomic position, peptide sequence, and binding affinity of all predicted neoantigens; the list of recurrent SV-neoantigens and their binding properties with high-prevalence HLA alleles; the intratumor heterogeneity of neoantigen per patient; the neoantigen burden (TNB and GANB) per patient; the tumor microenvironment subtype of each patient. [file 13059_2023_3005_MOESM2_ESM.docx]

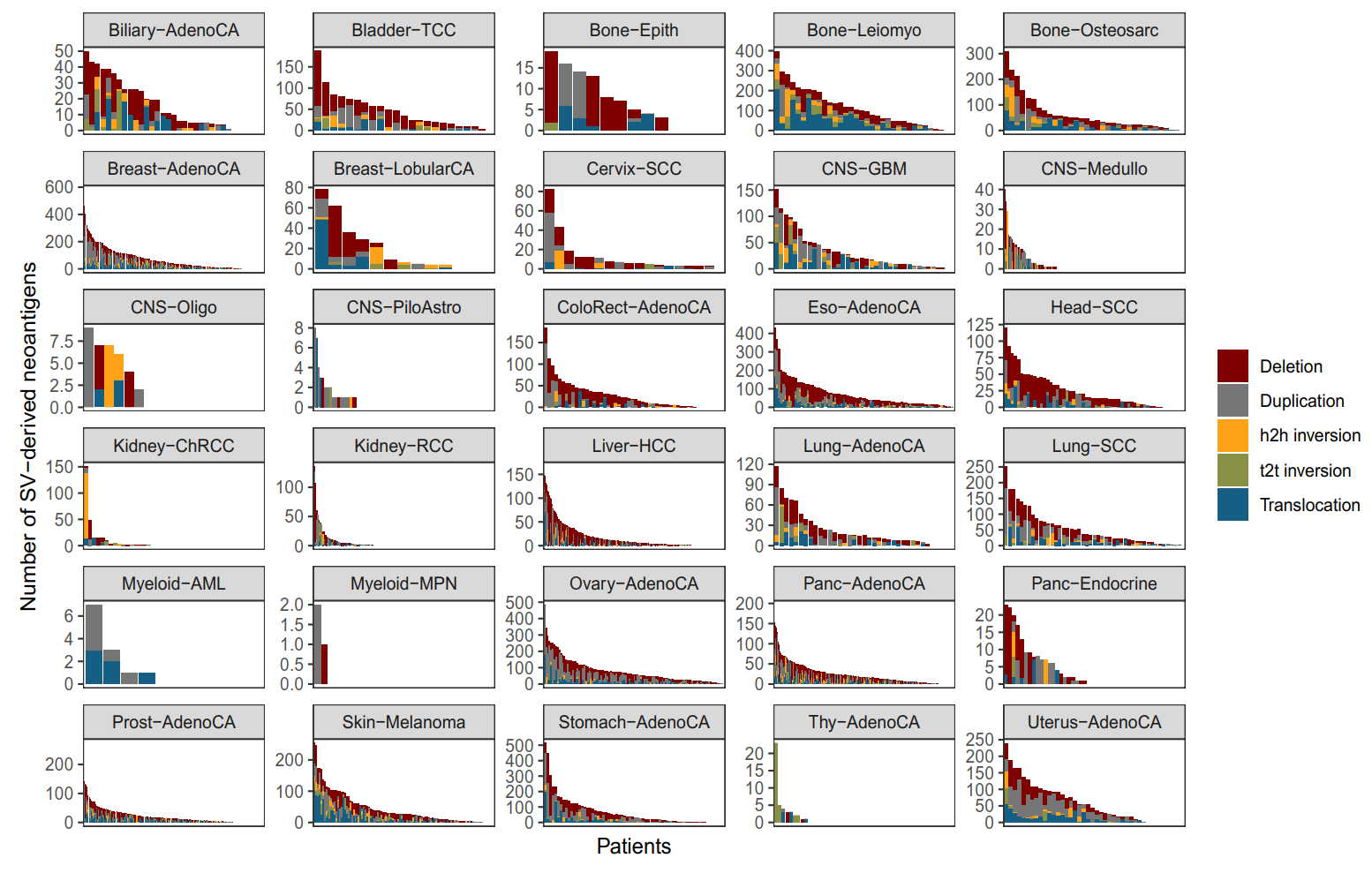


**Fig. S1 Landscape of SV-derived neoantigens in PCAWG.**

The number of SV-derived neoantigens per patient. Colors represent SV-derived neoantigenes from different types of SVs. Only cancer types with >10 tumors are included.


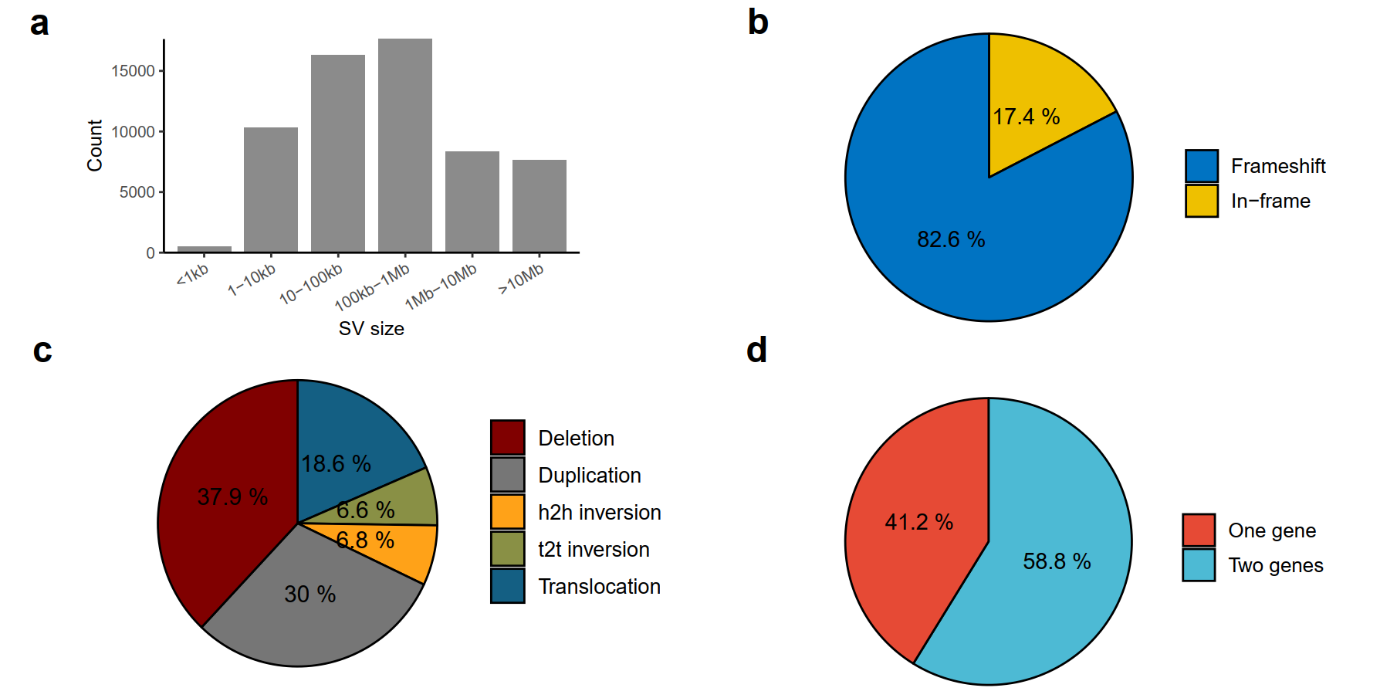


**Fig. S2 Genomic characteristics of SV-derived neoantigens.**

**a**, Number of SV-derived neoantigens categorized by the size of SVs. Only intra-chromosomal SVs are plotted. **b**, Percentage of frameshift and in-frame SV-derived neoantigens. **c**, Percentage of different SV types: deletion, duplication, translocation, head-to-head inversion (h2h inversion) and tail-to-tail inversion (t2t inversion). **d**, Percentage of SV-derived neoantigens created by SVs within one gene or SVs spanning two genes.


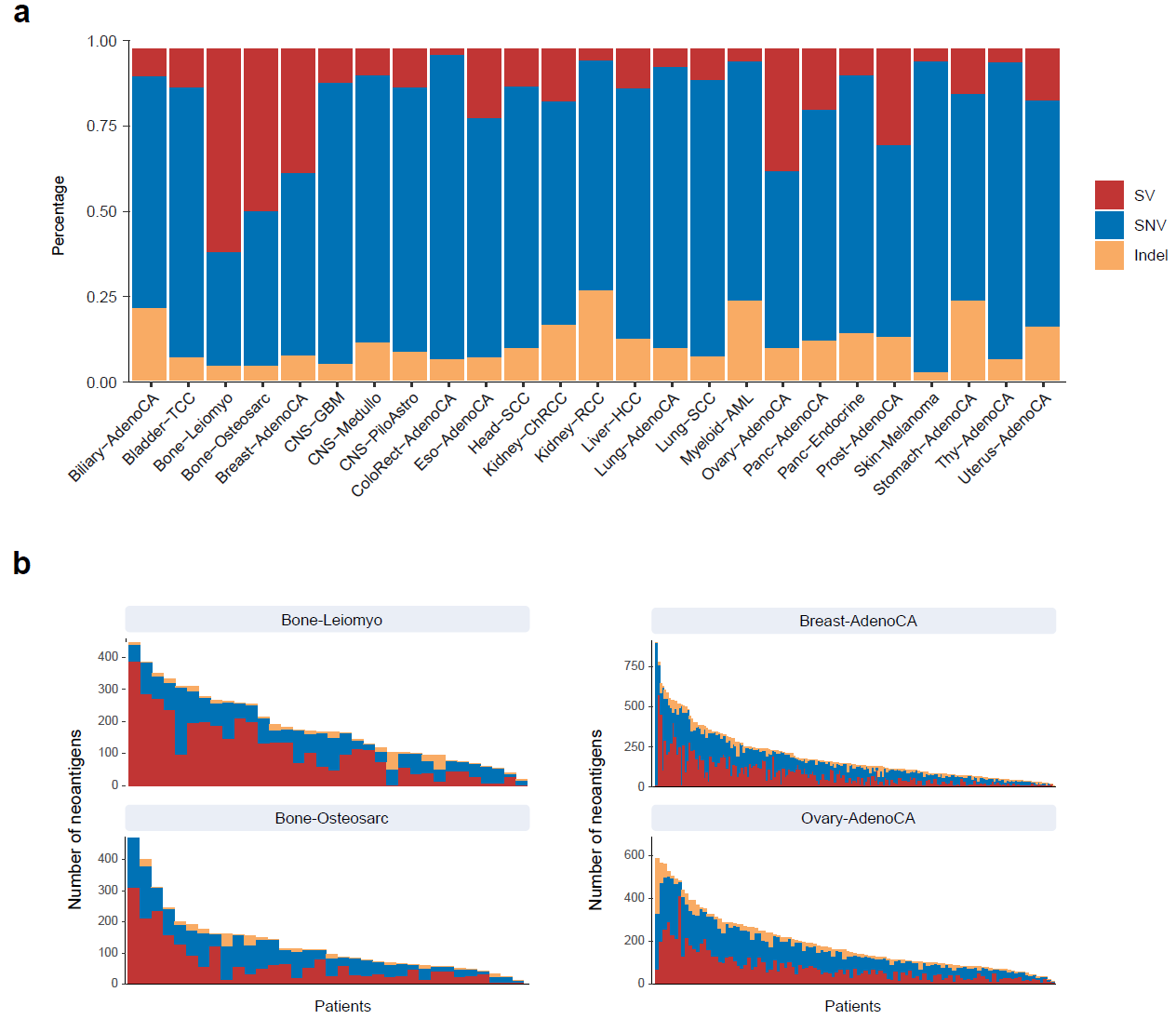


**Fig. S3 Contributions of SVs, SNVs and indels to the neoantigen repertoire.**

**a**, Proportions of neoantigens derived from SVs, SNVs and indels to the neoantigen repertoire of each cancer type. **b**, Number of neoantigens per patient categorized by their genomic sources.


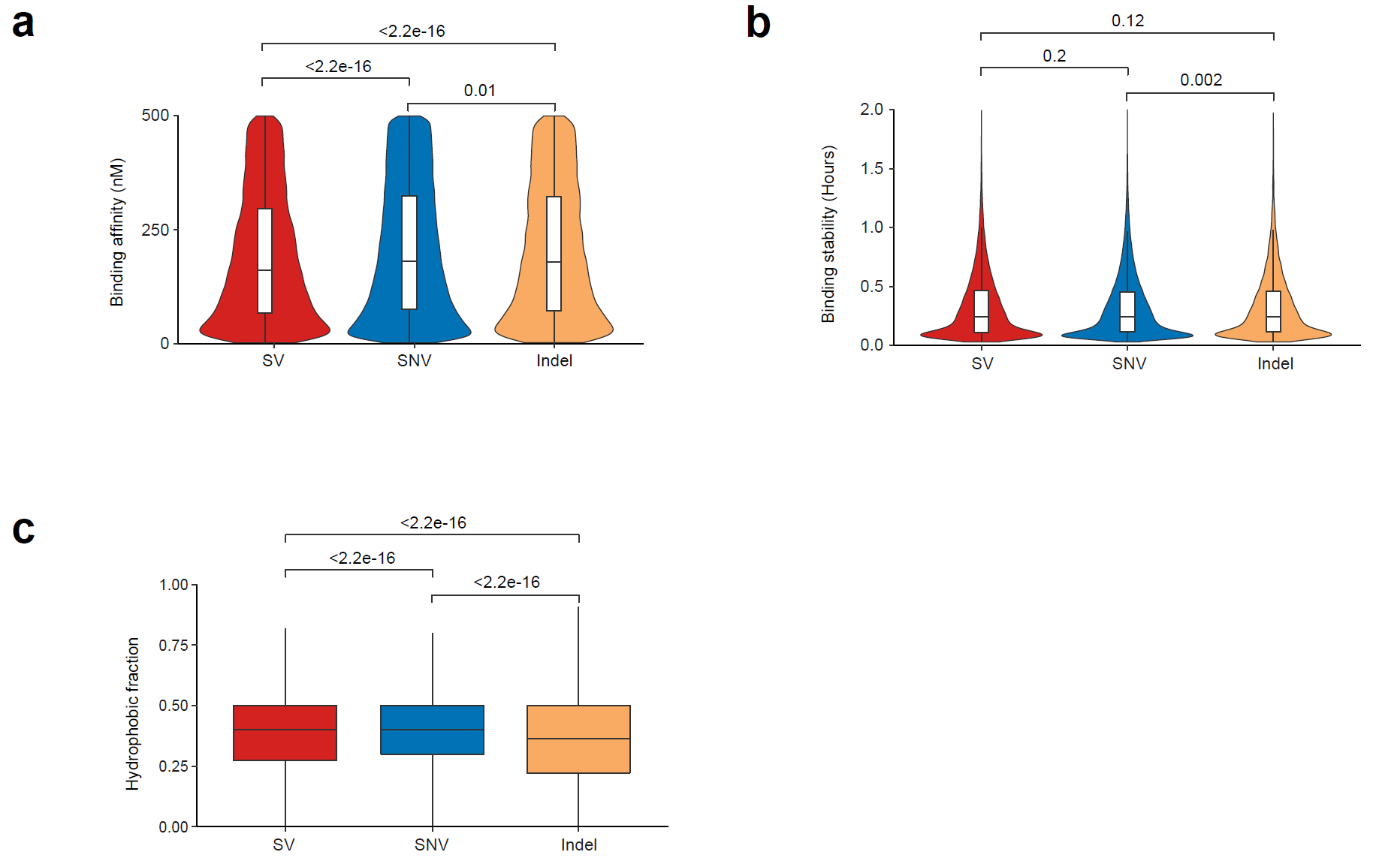


**Fig. S4 Comparison of basic neoantigen-related features across neoantigens from SVs, SNVs and indels.**

**a-c**, Differences in the binding affinity (**a**), binding stability (**b**) and hydrophobic fraction (**c**) across different neoantigen types (two-sided Wilcoxon rank-sum test). Boxplot hinges represent 25^th^ to 75^th^ percentiles, central lines represent median values; violin plot refers to the kernel probability density.


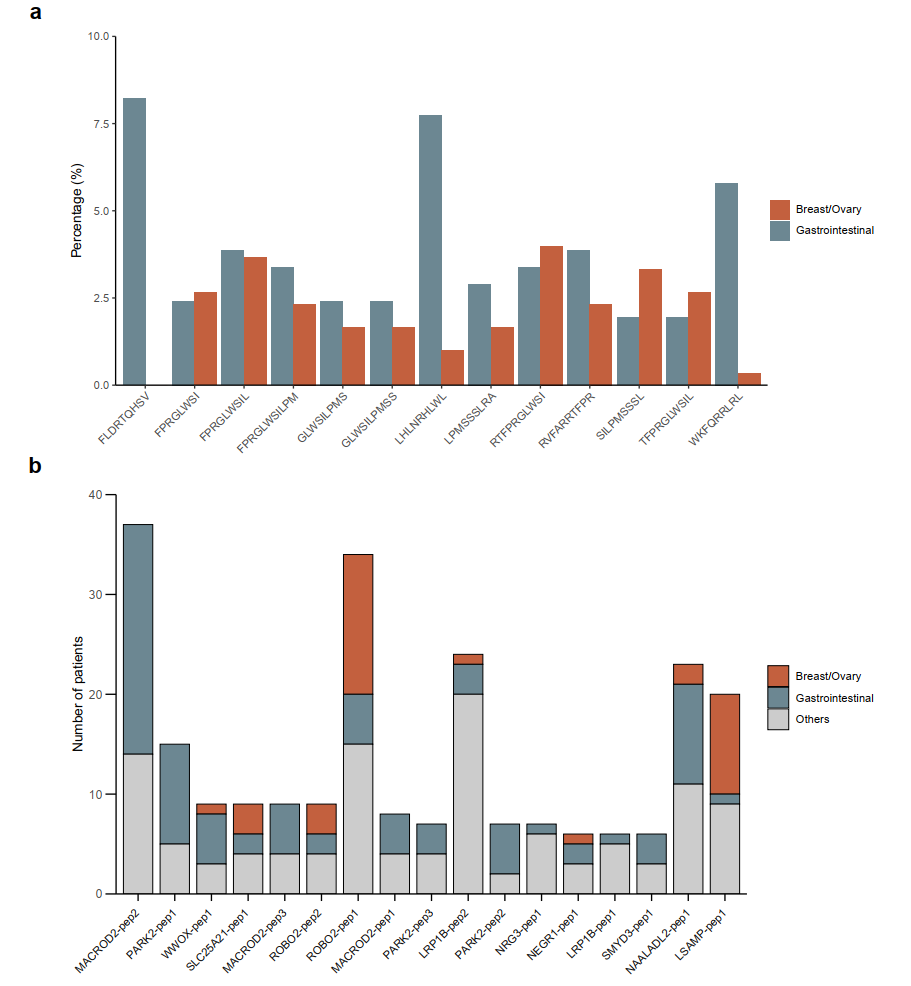


**Fig. S5 Shared SV-derived neoantigens.**

**a**, Prevalence of shared SV-derived neoantigens in gynecologic and gastrointestinal cancers. **b**, Number of patients bearing shared neo-peptides. Patients are colored by their cancer types (gynecologic, gastrointestinal or others).


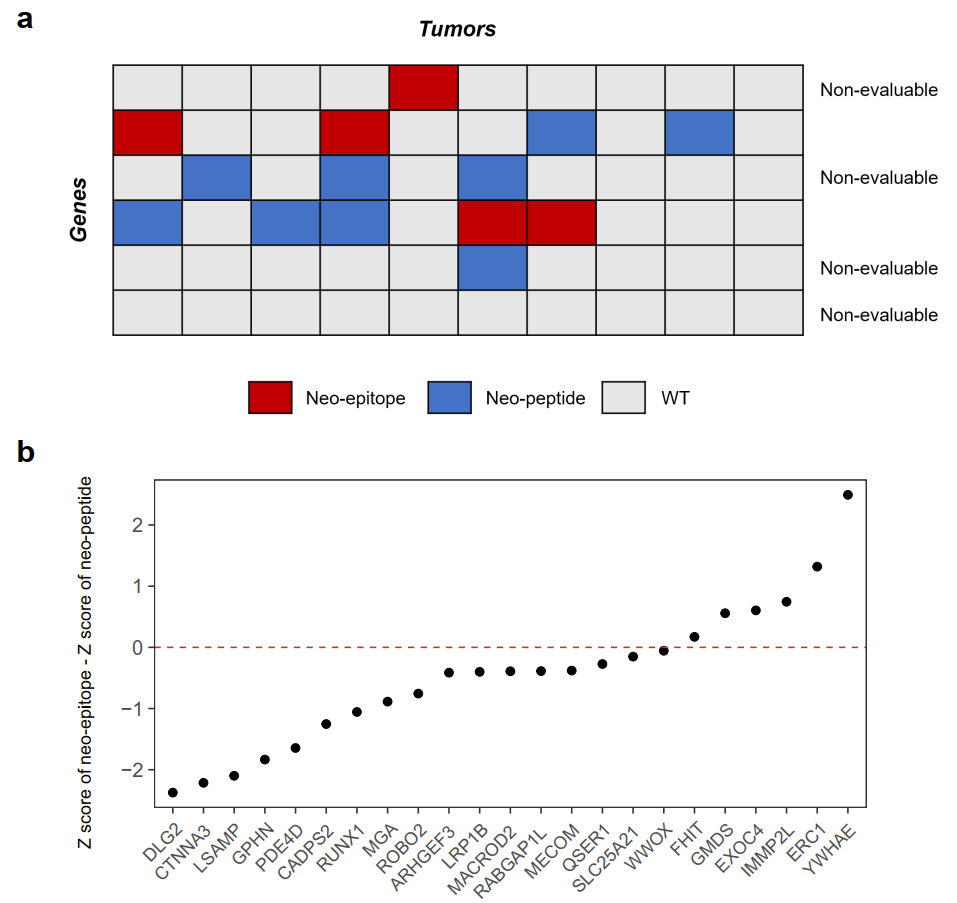


**Fig. S6 Diagram of the gene expression of neo-peptide and neoantigen-generating SVs.**

**a**, For each gene, it must have ≥2 patients harboring a same neo-peptide-generating SV, in which ≥1 patients can present it as neoantigen, and ≥1 patients cannot. Such genes are considered as bearing the same degree of NMD, thus are included for expression comparison (evaluable). Other genes are not included for this analysis (non-evaluable). **b**, Given a specific gene, the average Z-score (normalized expression within cancer type) of samples with neo-epitopes was subtracted by that of samples bearing the same neo-peptide, but could not be presented by MHC


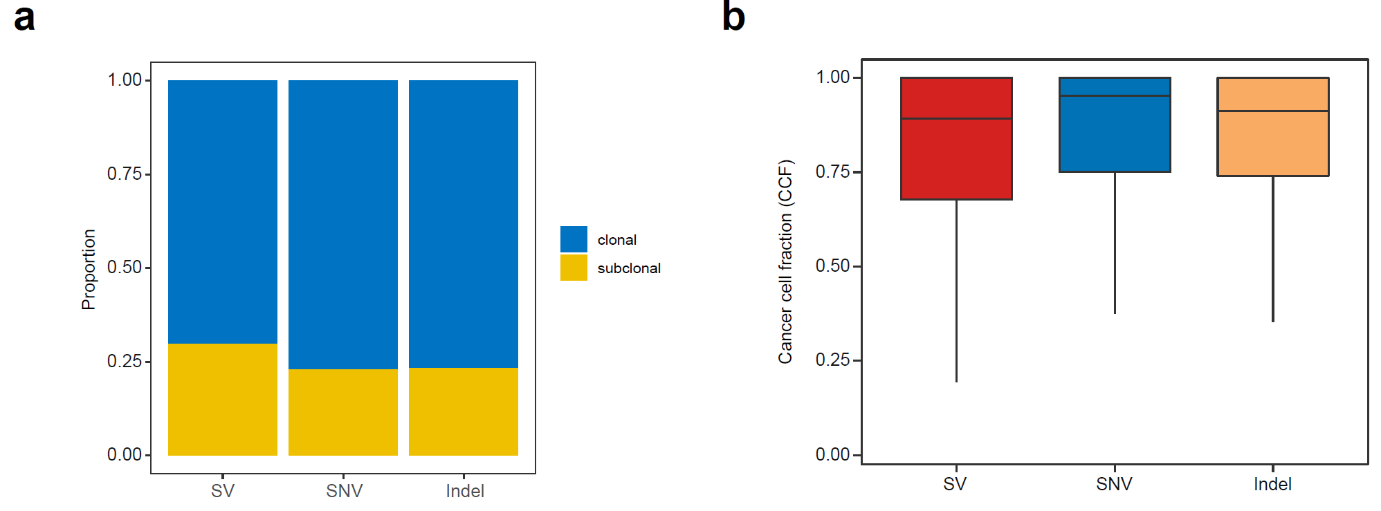


**Fig. S7 Intratumor heterogeneity (ITH) of the neoantigens derived from SVs, SNVs and indels**

**a**, Overall percentage of clonal/subclonal neoantigens from different alteration types. **b**, cancer cell fractions (CCF) of neoantigens from different alteration types. Boxplot hinges represent 25^th^ to 75^th^ percentiles, central lines represent median values.


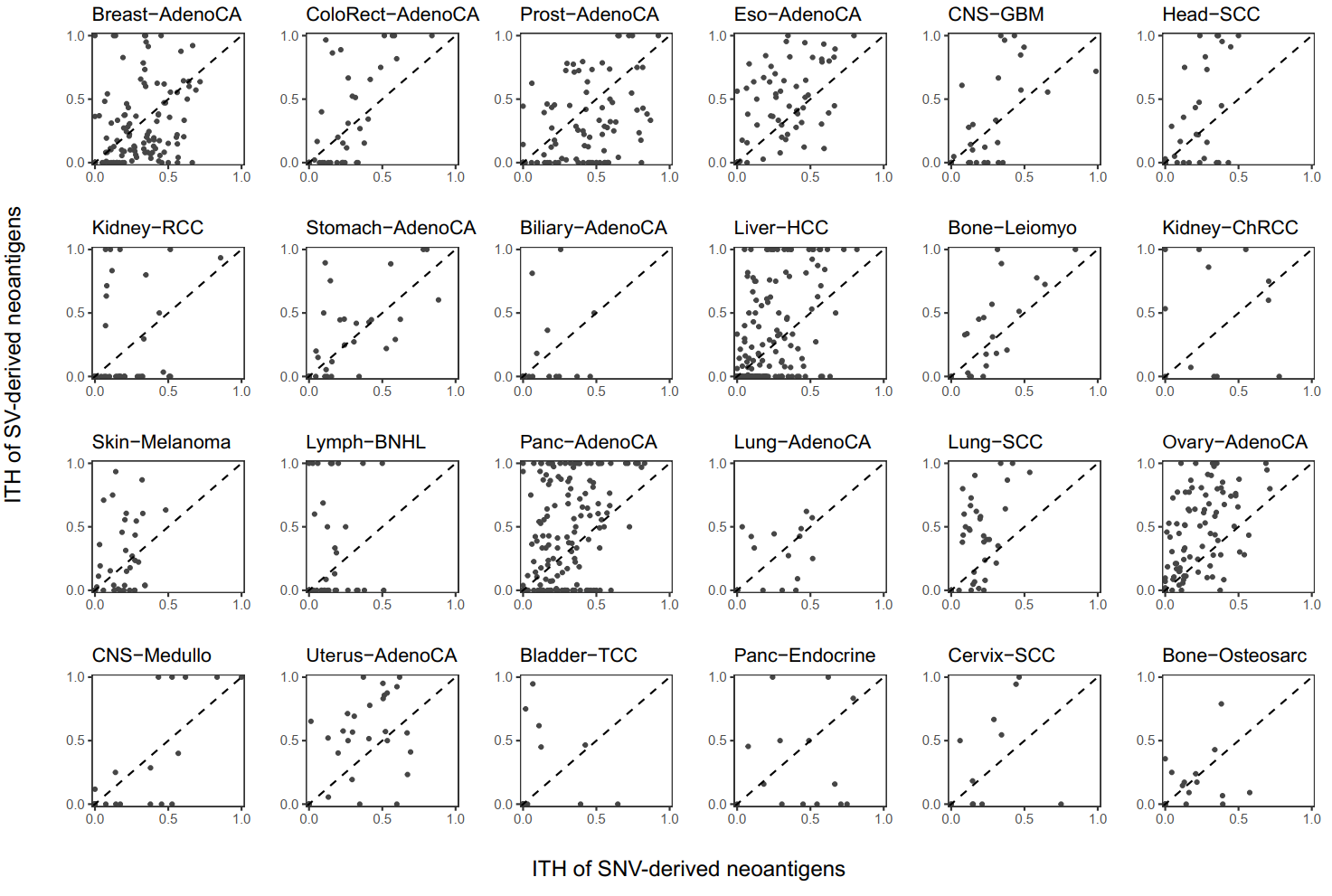


**Fig S8. ITH of neoantigens derived from SVs versus that from SNVs.**


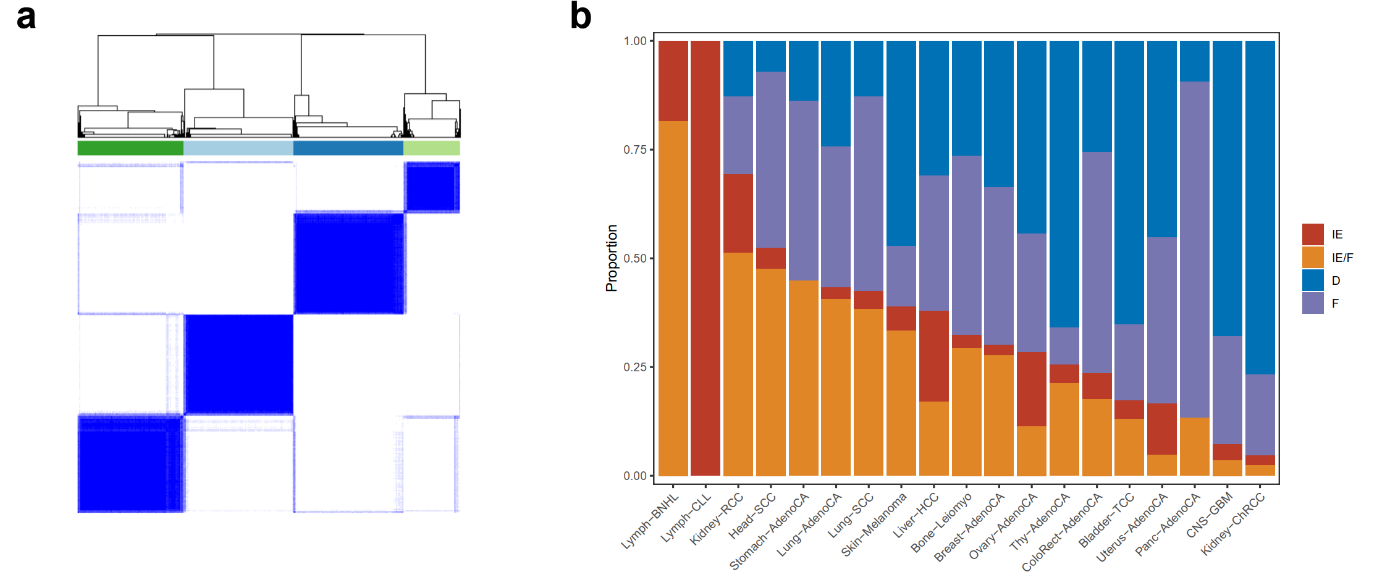


**Fig. S9 Consensus clustering of 1,188 tumor transcriptomes by 29 TME related signatures.**

**a**, Heatmap showing the consensus matrix of k-means clustering (k = 4). **b**, Proportion of 4 TME subtypes across cancer types. Cancer types are ordered by the sum of IE and IE/F subtypes. IE/F: immune-enriched, fibrotic; IE: immune-enriched, non-fibrotic; F: fibrotic; D: immune-depleted.


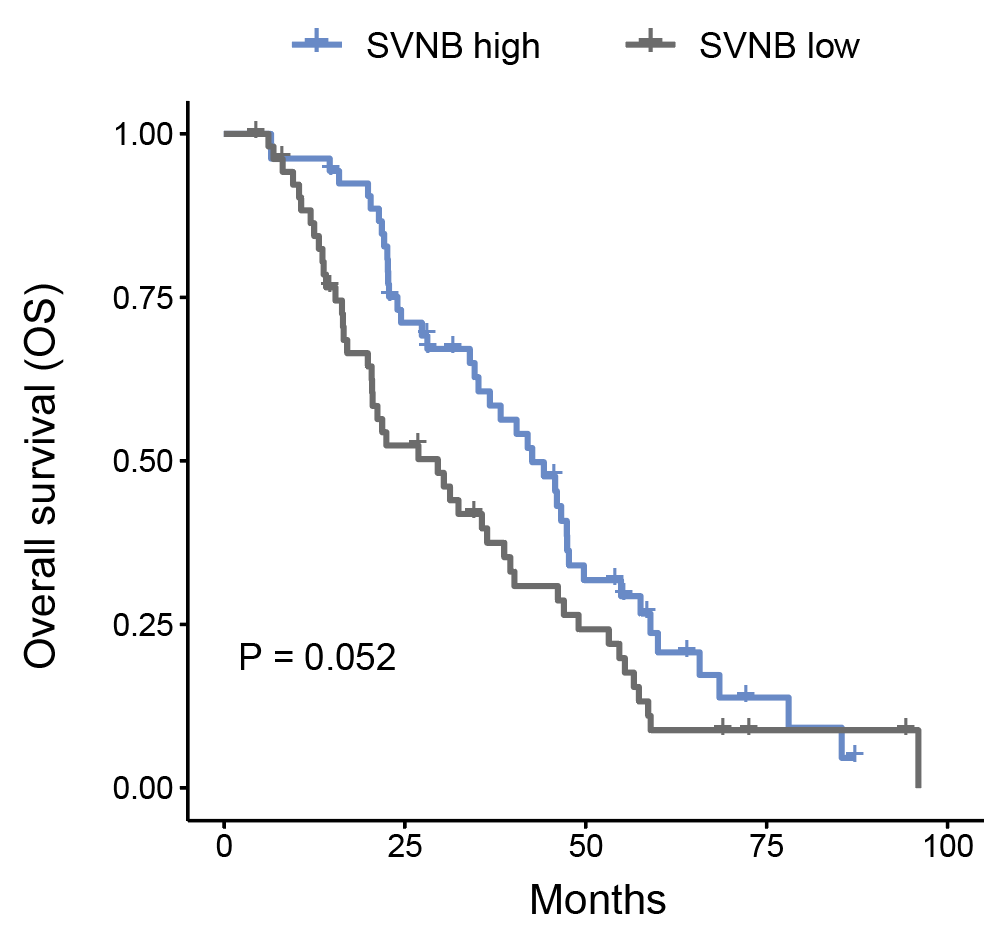


**Fig. S10 Kaplan-Meier curves of patients with ovary adenocarcinoma stratified by the median of SVNB.** Two-sided rank sum test was used to derive *P*-value.


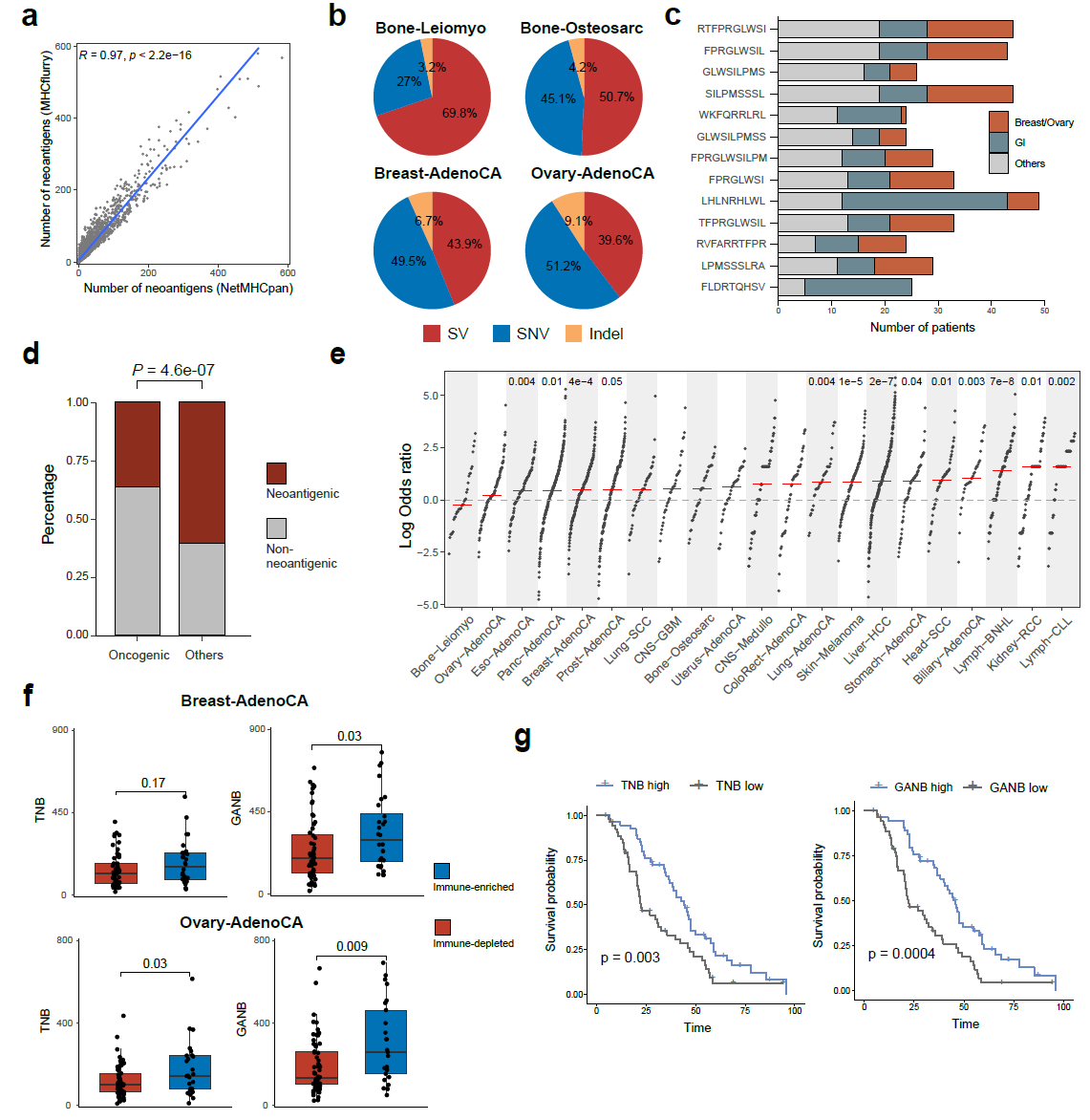


**Fig. S11 Validation of the main conclusions using MHCflurry.** (a) Scatterplot showing the correlation of neoantigen number predicted by NetMHCpan (x-axis) and by MHCflurry (y-axis). (b) Pie plot showing the relative percentage of SV, SNV, indel derived neoantigens. (c) SV-derived neoantigens listed in Fig. 3b and their associated cancer types. (d) Depleted neoantigenic SVs in oncogenes. (e) The odds ratios of generating neoantigens from subclonal and clonal SVs in different patients. Red bars indicate median values. (f) Differences in TNB/GANB between immune-enriched and immune-depleted tumors from Breast-AdenoCA and Ovary-AdenoCA. (g) Kaplan-Meier curves of patients with ovary adenocarcinoma stratified by TNB (left) and GANB (right), using median value as cutoff (two-sided rank sum test).
