# Supplementary material for: Comprehensive analysis of neoantigens derived from structural variation across whole genomes from 2528 tumors
Source: Genome Biol. 2023 Jul 17;24:169. doi: 10.1186/s13059-023-03005-9 (PMC10351168; doi:10.1186/s13059-023-03005-9)
Supplement: Supplementary file 3 — Additional file 3. Review history. [file 13059_2023_3005_MOESM3_ESM.docx]

Review History

**First round of review**

**Reviewer 1**

**Were you able to assess all statistics in the manuscript, including the appropriateness of statistical tests used?**

Yes, and I have assessed the statistics in my report.

**Comments to author:**

The authors investigate how structural variation in cancer genomes can lead to neoepitopes and, hence, could affect tumor evolution. They developed a computational approach where they leverage an existing method to predict MHC-binding to infer neoepitopes. They identify potential SV-derived neoantigens in the majority of investigated cancer types. The authors suggest that the presence of those neoantigens impacts immune infiltrates and survival. This obviously could have an impact for patient stratification for immunotherapy.

The main issue with the study is that the authors rely on predictions of immunogenicty / MHC binding, which might be inaccurate. There is no easy solution as validating those predictions would require laborious experimental validation, which is clearly out of scope of this manuscript. It's even more important then to validate the observations with follow-up analyses such as identifying signals of negative selection (as the authors already attempt to do) or functional consequences and address potential confounders there. I'm listing here a few points:

- The analysis shown in Fig 4a should be repeated on cancer type-level (for proteins and cancer types where there where a sufficiently high number of samples to perform this analysis)

- the authors should better disentangle positive and negative selection: could it be that the lack of SV-derived neoepitopes in oncogenes could be merely a consequence of those genes being in general protected from SVs? If I understand correctly the test the authors perform ("we found that in contrast to passengers, oncogenic SVs were less likely to generate neoantigens") they don't correct for number of residues affected, potential deleteriousness etc but these are important confounders that need to be taken into account.

- Do the authors observe a higher degree of subclonality for SV-derived neoepitopes as compared to all SV-derived peptides?

- "we proposed genomic alteration-related neoantigen burden (GANB), which integrated neoantigens from SNVs, indels and SVs together" (p8): it would be helpful to compare first the effect (with respect to immune infiltrates and survival) of the amount of neoantigens generated from SVs only to the amount of neoantigens generated from SNVs only (ie TNB) before integrating everything into the GANB score.

- how do the disease free survival curves corresponding to Fig 5g look like?

- as all analyses rely heavily on the netMHC predictions, I would reconfirm the main observations using an additional computational tool and / or experimentally determined MHC binding peptides (eg from IEDB)

Minor comments:

- A methods paragraph is missing in abstract. Like this it's hard to understand what the authors did from just reading the abstract

- "because somatic alterations are specific to tumor cells" - that is not true

- there are a number of typos, eg:

- p10: "the one with longest coding region was chose for analysis" -> "was chosen"

- p6: "was rare in pan-cancer cohort" -> missing "the"

**Reviewer 2**

**Were you able to assess all statistics in the manuscript, including the appropriateness of statistical tests used?**

Yes, and I have assessed the statistics in my report.

**Comments to author:**

Shi et al developed a new algorithm NeoSV to identify neoantigens generated by somatic SVs. The authors applied the algorithm on PCAWG data and performed a comprehensive analysis of the SV-driven neoantigens in many tumor types. The algorithm does a very good job to cover many different types of SV-driven neoantigens. The analyses of PCAWG data are well performed and yield interesting results. Such as, SV-driven neoantigens are better than SNV-derived ones, most neoantigens in sarcoma are SV-driven, some are shared by multiple patients, most of them are subclonal, GANB is a better measure than TNB. The main value of this study is that SV is not typically considered in neoantigen prediction. The algorithm certainly is of general interest and will be very useful for both research and perhaps even clinical use. However, there are a few critical issues with this study.

1. The authors introduce the article as if no one has ever done this type of work, which is not true. PMID 32206575 is a review article on this topic. Neoantimon and INTEGRATE-neo are two similar tools. The authors should benchmark their tool with.

2. It doesn't seem HLA typing was performed on PCAWG patients, which is essential in neoantigen prediction.

3. It seems SV were in silico transcribed. Whether mRNA molecules are produced or not is unclear. In fact, SVs are mostly disruptive to transcription. PCAWG samples have RNAseq data. This can easily be verified. The authors choose to consider the longest isoform which is also problematic. The actual isoform can be inferred from RNAseq data.

4. The negative selection section is interesting and makes good sense. However, I don't quite understand what fig4a is plotting. What is being compared? Same neo-peptides have different expression level. Is it because of different HLA alleles? Can statistical test be performed to derive a p value?

5. Some common neo-peptides can't be presented by MHC. Is it again because of HLA alleles? Does this also reflect negative selection?

6. PMID 30518613 showed noncoding regions are the main source of neoantigens. This should be discussed.

7. The discussion should not repeat what's been covered in results.

**Authors Response**

**Point-by-point responses to the reviewers’ comments:**

*We thank the reviewers for their insightful comments and suggestions. We list our detailed point-to-point responses as below. The comments are shown in blue and our responses shown in black.*

**Reviewer #1**

- **Overall:** The authors investigate how structural variation in cancer genomes can lead to neoepitopes and, hence, could affect tumor evolution. They developed a computational approach where they leverage an existing method to predict MHC-binding to infer neoepitopes. They identify potential SV-derived neoantigens in the majority of investigated cancer types. The authors suggest that the presence of those neoantigens impacts immune infiltrates and survival. This obviously could have an impact for patient stratification for immunotherapy.
  The main issue with the study is that the authors rely on predictions of immunogenicty / MHC binding, which might be inaccurate. There is no easy solution as validating those predictions would require laborious experimental validation, which is clearly out of scope of this manuscript. It's even more important then to validate the observations with follow-up analyses such as identifying signals of negative selection (as the authors already attempt to do) or functional consequences and address potential confounders there. I'm listing here a few points.

***Response****: We sincerely thank you for your constructive comments and valuable suggestions to help us improve this paper. The questions are addressed below.*

- **Comment 1**: The analysis shown in Fig 4a should be repeated on cancer type-level (for proteins and cancer types where there where a sufficiently high number of samples to perform this analysis).

***Response****: Thanks. We agree with the referee that the analysis should also be performed on cancer type-level. However, since we only compared the gene expression of samples harboring the same neo-peptides, it can be expected that the samples meeting this criterion are extremely limited. Actually, except LSAMP, all other genes have less than 10 available samples, which makes the cancer-type level analysis unfeasible. This is just the reason that we used a cautious statement here: “…SV-derived neoantigens probably were subject to modest expression reduction…” (page 5, line 209).*

- **Comment 2**: The authors should better disentangle positive and negative selection: could it be that the lack of SV-derived neoepitopes in oncogenes could be merely a consequence of those genes being in general protected from SVs? If I understand correctly the test the authors perform ("we found that in contrast to passengers, oncogenic SVs were less likely to generate neoantigens") they don't correct for number of residues affected, potential deleteriousness etc but these are important confounders that need to be taken into account.

***Response****: Thanks. We agree that the lack of SV-derived neoepitopes in oncogenes could be due to oncogenes being protected from SVs. However, our analysis showed that oncogenes usually bore more SVs than passengers (Fig. R1), which implied that the depletion of neoantigens in oncogenes could not be merely attributed to the lack of SVs. Moreover, in Fig. 4b, instead of comparing the number of neoantigen-generating SVs per gene between oncogenes and other genes, we compared the proportions of neoantigen-generating SVs between oncogenic SVs and passenger SVs. Fisher’s exact test showed that the proportion of neoantigen-generating SVs in oncogenic SVs was significantly smaller than that in passenger SVs (odds ratio significantly less than 1). This depletion of neoantigen-generating SVs in oncogenic SVs was more likely to be a result of negative selection by immune system.*

**Fig. R1** Boxplot showing the difference in number of SVs per gene between oncogenes and passengers (Two-sided Wilcoxon rank sum test).

*To further account for the confounding factors,* *we considered the following logistic regression model:*

$$logit\left( P \right)=\beta_{0}+\beta_{1}X_{1}+\beta_{2}X_{2}+\beta_{3}X_{3}$$

*where* $P$ *represents the probability of an SV generating a neoantigen,* $X_{1}$ *represents whether this SV is oncogenic,* $X_{2}$ *represents the number of affected residues and* $X_{3}$ *represents whether it is frameshift. Because unlike SNVs, there was no well-known deleteriousness scores for SVs, here, we directly used the impact on open reading frames to represent the “deleteriousness” of SVs. The estimates of coefficients are shown in the table below:*

|  | **Estimate** | **Standard error** | ***P-*value** |
| --- | --- | --- | --- |
| Intercept ($\beta_{0}$) | 0.031 | 0.035 | 0.366 |
| Oncogenic ($\beta_{1}$) | -1.004 | 0.183 | 3.79e-08 |
| Residues ($\beta_{2}$) | 0.051 | 0.009 | 0.120 |
| Frameshift ($\beta_{3}$) | 0.459 | 0.024 | < 2.2e-16 |

*We found that, even after controlling the confounding variables, oncogenic SVs still had a significantly decreased probability of generating neoantigens, as indicated by the significant negative value of* $\beta_{1}$*. We also found that frameshift SVs were more likely to be neoantigenic, which was reasonable and expected. This analysis has been added to the revised manuscript (page 5, line 218 and Supplementary Table S6)*

- **Comment 3**: Do the authors observe a higher degree of subclonality for SV-derived neoepitopes as compared to all SV-derived peptides?

***Response****: To answer this question, we compared the subclonality of SV-derived neoepitopes and SV-derived neo-peptides within each cancer type. As shown in Fig. R2, most dots locate alongside the diagonal line, suggesting minimal difference between the subclonality of SV-derived neoepitopes and SV-derived neo-peptides.*

**Fig. R2** Scatterplot showing the proportion of subclonal neo-peptides and subclonal neo-epitopes. Each dot represents a tumor sample.

- **Comment 4**: "we proposed genomic alteration-related neoantigen burden (GANB), which integrated neoantigens from SNVs, indels and SVs together" (p8): it would be helpful to compare first the effect (with respect to immune infiltrates and survival) of the amount of neoantigens generated from SVs only to the amount of neoantigens generated from SNVs only (ie TNB) before integrating everything into the GANB score.

***Response****: Thanks for the great suggestion. We analyzed SV-derived neoantigens separately to help better understanding the contributions of SVs in GANB, and defined the SV-derived neoantigen burden (SVNB). We found that, (1) the differences in SVNB were larger between immune-enriched and immune-depleted tumors than the differences in TNB for several cancer types, especially for breast and ovarian cancer (Fig. R3a), which explained the significant better performance of GANB than TNB in these two cancer types. (2) SVNB could also predict survival in ovarian cancer (Fig. R3b), though the P-value (0.052) was less significant than GANB and TNB. These results were also added to Fig. 5d and Supplementary Fig.10.*

**Fig. R3** **The association of SV-derived neoantigen burden (SVNB) with TME and survival**. (a) Heatmap showing the difference in TNB (left), GANB (middle) and SVNB (right) between immune-enriched tumors and immune-depleted tumors (two-sided Wilcoxon rank-sum test). Red indicates higher neoantigen load in immune-enriched tumors while blue indicates higher neoantigen load in immune-depleted tumors. **P*<0.05, ***P*<0.005. (b) Kaplan-Meier curves of patients with ovary adenocarcinoma stratified by the median of SVNB.

- **Comment 5**: how do the disease free survival curves corresponding to Fig 5g look like?

***Response****: It seems that PCAWG does not have disease free survival or progression free survival data in its released clinical data (*[*https://dcc.icgc.org/releases/PCAWG/clinical_and_histology*](https://dcc.icgc.org/releases/PCAWG/clinical_and_histology)*). We thus could not perform this analysis.*

- **Comment 6**: as all analyses rely heavily on the netMHC predictions, I would reconfirm the main observations using an additional computational tool and / or experimentally determined MHC binding peptides (eg from IEDB).

***Response****: Thanks for this great suggestion. To further confirm the reliability of our conclusions, we used another widely-used algorithm-MHCflurry. MHCflurry incorporates mass spectrometry-based experimental data to improve the prediction of the MHC binding^1^. We found that the number of SV-derived neoantigens per patient predicted by MHCflurry has strong correlation with that predicted by NetMHCpan (Fig. R4a). Our main observations could also be repeated using the results from MHCflurry:*

1. *Similar to the NetMHCpan results (Fig. 2d), SV-derived neoantigens contribute to a large proportion of the neoantigen repertoire of bone leiomyoma (69.8%), bone osteosarcoma (50.7%), breast adenocarcinoma (43.9%) and ovary adenocarcinoma* *(39.6%) (Fig. R4b).*
2. *Shared neoantigens listed in Fig. 3b could be successfully predicted by MHCflurry. In addition, they are also enriched in breast, ovarian and gastrointestinal cancers (Fig. R4c).*
3. *Compared to passengers, oncogenic SVs are less likely to generate neoantigens (60.6% vs 34.8%, P = 4.6e-07) (Fig. R4d). This is to repeat the result in Fig. 4b.*
4. *Subclonal SVs are more likely to create neoantigens than clonal SVs (Fig.R4e). This is to repeat the result in Fig. 4f.*
5. *In breast and ovarian cancer, the differences in GANB between immune-enriched and immune-depleted tumors are larger than the differences in TNB (Fig.R4f). This is to repeat the result in Fig. 5e*
6. *GANB has better performance than TNB in predicting survival of breast cancer (Fig.R4g). This is to repeat the result in Fig. 5g.*

*Considering the high consistency of above results, we believe our conclusions in the manuscript are reliable and not dependent on specific algorithm. These results have been added to the revised manuscript (page 9, line 395):*

*“Third, our analysis relied on the in silico MHC binding prediction. The conclusions in the paper were based on the NetMHCpan and could also be repeated using another well-known MHC binding prediction algorithm MHCflurry (Supplementary Fig. 11). However, considering the discrepancies between in silico predictions and experimental results, whether these predicted neoantigens could elicit T cell responses need further experimental validation.”*

*In addition, we incorporated MHCflurry to the new release (v0.0.3) of our workflow NeoSV (https://github.com/ysbioinfo/NeoSV), to provide more options to investigators.*

**Fig. R4** **Validating main conclusions in the manuscript using MHCflurry.** (a) Scatterplot showing the correlation of neoantigen load predicted by NetMHCpan (x-axis) and by MHCflurry (y-axis). (b) Pie plot showing the relative percentage of SV, SNV, indel derived neoantigens. (c) Shared SV-derived neoantigens listed in Fig. 3b and their associated cancer types. (d) Neoantigenic SVs depleted in oncogenic SVs. (e) The odds ratios of generating neoantigens from subclonal and clonal SVs in different patients. Red bars indicate median values. (f) Differences in TNB/GANB between immune-enriched and immune-depleted tumors from Breast-AdenoCA and Ovary-AdenoCA. (g) Kaplan-Meier curves of patients with ovary adenocarcinoma stratified by TNB (left) and GANB (right), using median value as cutoff.

- **Comment 7**: a methods paragraph is missing in abstract. Like this it's hard to understand what the authors did from just reading the abstract.

***Response****: Thanks for this kind advice. We added more descriptions of our workflow to the abstract: “Here, we developed a computational method termed NeoSV, which incorporated SV annotation, protein fragmentation and MHC binding prediction together, to predict SV-derived neoantigens”. In addition, we have described the details of this workflow in Results (page 3, line 96) and Methods (page 9, line 381) to help readers get a better understanding.*

- **Comment 8**: "because somatic alterations are specific to tumor cells" - that is not true.

***Response****: We removed this sentence in the revised manuscript and changed it to: “Meanwhile, these tumor-specific neoantigens are neither subject to central immune tolerance nor likely to cause autoimmunity” (page 2, line 47).*

- **Comment 9**: there are a number of typos, eg: p10: "the one with longest coding region was chose for analysis" -> "was chosen". p6: "was rare in pan-cancer cohort" -> missing "the".

***Response****: We apologize for these typos and thank the reviewer for the his/her careful reading. We corrected these typos and double-checked the paper.*

**Reviewer #2**

- **Overall**: Shi et al developed a new algorithm NeoSV to identify neoantigens generated by somatic SVs. The authors applied the algorithm on PCAWG data and performed a comprehensive analysis of the SV-driven neoantigens in many tumor types. The algorithm does a very good job to cover many different types of SV-driven neoantigens. The analyses of PCAWG data are well performed and yield interesting results. Such as, SV-driven neoantigens are better than SNV-derived ones, most neoantigens in sarcoma are SV-driven, some are shared by multiple patients, most of them are subclonal, GANB is a better measure than TNB. The main value of this study is that SV is not typically considered in neoantigen prediction. The algorithm certainly is of general interest and will be very useful for both research and perhaps even clinical use. However, there are a few critical issues with this study.

***Response****: We appreciate the reviewer’s encouraging comments and helpful suggestions which help us improve this work. The questions are addressed as follows.*

- **Comment 1**: The authors introduce the article as if no one has ever done this type of work, which is not true. PMID 32206575 is a review article on this topic. Neoantimon and INTEGRATE-neo are two similar tools. The authors should benchmark their tool with.

***Response****: Thanks for pointing out these literatures. In the Introduction (page 2, line 84), we added description on previous studies related to SV-derived neoantigens, including PMID 32206575 which showed the neoantigenic potential of SV in mesothelioma: “Recently, SV-derived neoantigens has been reported in mesothelioma and demonstrated to be immunogenic in head and neck cancer”. As for the two similar tools, we discussed their similarities and differences compared with our method in the Discussion section and benchmarked Neoantimon, which were briefly summarized below:*

- ***INTEGRATE-neo****:INTEGRATE-neo only predicts neoantigens from gene fusions and does not consider other types of SVs such as SVs within one gene. According to our analysis in Fig. 1e, ~41.2% SV-derived neoantigens are from SVs involving only one gene, all of which are missed by INTEGRATE-neo. Thus, this tool is very different from ours and we do not benchmark it. This discussion has been added to the Discussion section (page 8, line 336).*
- ***Neoantimon****: Neoantimon is very similar to our tool, with only 3 minor differences. First, Neoantimon requires pre-annotated SVs as input, while our tool is a one-stop solution which accepts raw VCF (variant calling format) file, thus is more convenient for users. Second, Neoantimon discards read-through SVs (missing the stop codon and leading to the translation of 3’-untranslated region), while our tool includes these SVs with a specific “read-through” label. Third, Neoantimon relies on the UCSC database for annotation, while our tool depends on the Ensembl database. To compare, we also ran Neoantimon on the PCAWG data and found that its neoantigen predictions had a high degree of consistency with our predictions (Fig. R5). The non-overlapping part was mainly attributed to the difference between the UCSC and Ensembl database and the read-though SVs. We added these descriptions and benchmarking results in the Discussion section (page 8, line 327).*

**Fig. R5** Venn diagram showing the intersection of SV-derived neoantigens predicted by NeoSV and Neoantimon.

- **Comment 2**: It doesn't seem HLA typing was performed on PCAWG patients, which is essential in neoantigen prediction.

***Response****: We apologize for missing the reference. The HLA types of PCAWG have been inferred using the ALPHLARD algorithm in a previous work^2^. We added this citation in the Methods section of the revised manuscript.*

- **Comment 3**: It seems SV were in silico transcribed. Whether mRNA molecules are produced or not is unclear. In fact, SVs are mostly disruptive to transcription. PCAWG samples have RNAseq data. This can easily be verified. The authors choose to consider the longest isoform which is also problematic. The actual isoform can be inferred from RNAseq data.

***Response****: Following the reviewer’s suggestion, we verified SV predictions using RNA-seq data. Since downloading all raw RNA-seq data from PCAWG will take several months, we selected 50 breast cancer samples with high neoantigenic SV load (median = 32) and downloaded the FASTQ files of their RNA-seq data. For each SV, we created a 200bp SV-sequence by concatenating the 100bp upstream sequence of its 5’ breakpoint and the 100bp downstream sequence of its 3’ breakpoint. Then, for each cancer sample, we added all its SV-sequences to the reference transcriptome sequences and aligned the short reads of RNA-seq to this reference transcriptome. If the breakpoint of an SV-sequence is covered by more than 2 short reads, we consider the SV as validated by RNA-seq.*

*Our analysis showed that ~58% neoantigenic SVs could be validated by RNA-seq (Fig. R6), which was a reasonably high validation rate. In addition, these validation rates might underestimate the true positive rates of the SV predictions. First, the read length of these sample is only 50bp, making it more difficult to cover the breakpoints. Second, the sensitivity of RNA-seq heavily depends on the abundance of a transcript. However, transcripts with low expression can also be functionally important. For neoantigens, a recent review paper^3^ also pointed out that neoantigens with low expression can also induce anti-tumor responses and is important for immunotherapy.*

*We agree with the reviewer that using the longest isoform is not ideal. However, inferring isoform from RNA-seq is very challenging^4^. The major hurdle is the read length of the next-generation sequencing data. Although split reads can be used to detect individual splice junctions, it is difficult to accurately assemble them to a full transcript and determine the abundance of each isoform. As a consequence, when analyzing alternative splicing events based on RNA-seq, most studies (e.g., the TCGA marker paper on alternative splicing^5^) usually focused on individual exon/intron, but not on the whole transcript. Therefore, the isoform with the longest CDS is commonly used as an alternative solution. The third generation sequencing with long reads (usually >10kb) can overcome this limitation. We added these discussions in the Discussion section of the revised manuscript (page 8, line 348). In addition, we also added an option “--transcript-list” in the new release (v0.0.3) of our tool (https://github.com/ysbioinfo/NeoSV), which enabled using a user-defined isoform list for neoantigen prediction.*

**Fig. R6** Barplot showing the proportion of neoantigenic SVs supported by RNA-seq across 50 breast cancer samples. Dashed line represents the median proportion.

- **Comment 4**: The negative selection section is interesting and makes good sense. However, I don't quite understand what fig4a is plotting. What is being compared? Same neo-peptides have different expression level. Is it because of different HLA alleles? Can statistical test be performed to derive a p value?

***Response****: We apologize for the confusion. In Fig. 4a, given a shared neo-peptide generated from SVs of a gene, we took all patients with this neo-peptide and divide them into two groups. In the neo-epitope group, the neo-peptide can be presented by the MHC of the patients. In the neo-peptide group, the neo-peptide cannot be presented by the MHC of the patients. Then, we compared the gene expression between the neo-epitope group and neo-peptide group. Taking LSAMP as an example: 11 patients had the same neo-peptide generated by a LSAMP deletion, while only 7 of them could present this peptide as neo-epitopes, because the patients had different HLA alleles. Therefore, the neo-epitope group had 7 patients and the neo-peptide group had 4 patients. Then we calculated the ratio between the median expressions of the neo-epitope and neo-peptide groups and plotted the ratio (0.475) on the y-axis of Fig. 4a.*

*Following the reviewer’s suggestion, we derived an overall P-value based on the one-sample Wilcoxon rank sum test. The null hypothesis of the test was that the median of the expression ratios of these genes was 1. The P-value was 0.00013. It further confirmed our conclusion: the negative selection from immune cells led to reduced expression of the genes bearing SV-derived neoantigens.*

- **Comment 5**: Some common neo-peptides can't be presented by MHC. Is it again because of HLA alleles? Does this also reflect negative selection?

***Response****: The reviewer’s understanding is exactly what our results indicated: tumor cells bearing neoantigenic SVs were more likely to be eliminated by the immune system. Therefore, after the negative selection, the common SVs (or neo-peptides) that could be observed tend to be non-neoantigenic. HLA type had a big impact on this selection process. Similar results have also been observed for SNV-derived neoantigens^6^.*

- **Comment 6**: PMID 30518613 showed noncoding regions are the main source of neoantigens. This should be discussed.

**Response**: Thanks for pointing out this reference. We added discussions about the neoantigens from noncoding regions in the Discussion section (page 8, line 343): “a recent study pointed out that noncoding regions can be a major source of neoantigens, suggesting the importance of intergenic SVs in neoantigen prediction. However, because of the computational challenge of gene boundary prediction, we did not consider SVs in intergenic regions. Future improvements on “top-down” strategies like MHC-binding peptide mass spectrometry could help to better study these uncanonical neoantigens”.

- **Comment 7**: The discussion should not repeat what's been covered in results.

***Response****: Thanks for this kind suggestion. We added more discussions about the potential limitations of this work and future directions (page 8, line 343).*

***References***

*1	O'Donnell, T. J., Rubinsteyn, A. & Laserson, U. MHCflurry 2.0: Improved Pan-Allele Prediction of MHC Class I-Presented Peptides by Incorporating Antigen Processing. Cell Syst* ***11****, 418-419, doi:10.1016/j.cels.2020.09.001 (2020).*

*2	Yang, F. et al. Quantifying immune-based counterselection of somatic mutations. PLoS Genet* ***15****, e1008227, doi:10.1371/journal.pgen.1008227 (2019).*

*3	Lang, F., Schrors, B., Lower, M., Tureci, O. & Sahin, U. Identification of neoantigens for individualized therapeutic cancer vaccines. Nat Rev Drug Discov* ***21****, 261-282, doi:10.1038/s41573-021-00387-y (2022).*

*4	Steijger, T. et al. Assessment of transcript reconstruction methods for RNA-seq. Nat Methods* ***10****, 1177-1184, doi:10.1038/nmeth.2714 (2013).*

*5	Kahles, A. et al. Comprehensive Analysis of Alternative Splicing Across Tumors from 8,705 Patients. Cancer Cell* ***34****, 211-224 e216, doi:10.1016/j.ccell.2018.07.001 (2018).*

*6	Marty, R. et al. MHC-I Genotype Restricts the Oncogenic Mutational Landscape. Cell* ***171****, 1272-1283 e1215, doi:10.1016/j.cell.2017.09.050 (2017).*

**Second round of review**

**Reviewer 1**

While the revisions improved the manuscript, some doubts remain:
The authors repeat their analyses with another tool predicting MHC binding (MHCflurry). While they can reconfirm their results, the results look very similar. I assume this is because the predictions from the two tools are mostly the same? It would substantially strengthen the results to have a complementary validation of the observations. I would suggest to either show that the overlap between the tools of the predicted peptides per patient is relatively low (ie not more than 60% / 70% of overlapping regions) and hence this alternative approach is indeed an independent validation of the observations of the authors. Or, alternatively, to repeat the analyses with a really independent approach of defining MHC binding peptides (eg using the experimentally determined ones from IEDB).
In a similar line, the author's reply to my request to adapt Figure 4A to perform the analysis on cancer type level, does not convince me. They argue that for most genes they have only small number of samples with neo-peptides and neo-epitopes per cancer types and hence the analysis is not feasable on cancer type level. I get this point but it is very risky to compute the expression ratios pooling a large number of tissues (given that the number and tissue composition of neo-peptides and neo-epitopes largely differs, which can easily create confounders). At least they should control for tissue composition when computing the ratios (ie subsample neo-epitopes / -peptides in order to have expression values from the exact same composition of tissues in the numerator and denominator).

**Reviewer 2**

All my concerns have been properly addressed.

**Authors Response**

**Point-by-point responses to the reviewers’ comments:**

Reviewer 1:
Comment 1: The authors repeat their analyses with another tool predicting MHC binding (MHCflurry). While they can reconfirm their results, the results look very similar. I assume this is because the predictions from the two tools are mostly the same? It would substantially strengthen the results to have a complementary validation of the observations. I would suggest to either show that the overlap between the tools of the predicted peptides per patient is relatively low (ie not more than 60% / 70% of overlapping regions) and hence this alternative approach is indeed an independent validation of the observations of the authors. Or, alternatively, to repeat the analyses with a really independent approach of defining MHC binding peptides (eg using the experimentally determined ones from IEDB).

*Response: Thanks for this kind advice. In fact, the neoantigens predicted by MHCflurry have a median of 63.4% overlap with those predicted by NetMHCpan (Fig. R1a in the "Response letter" docx file), thus could be regarded as a complementary validation. To further confirm our result, we used TransMut (Chu et al., 2022, Nat Mach Intell, 4, 300-311), a recently developed tool that is more sensitive for MHC binding prediction. We found that although there are only 26.5% overlap between TransMut and NetMHCpan (Fig. R1b in the "Response letter" docx file), the main conclusions could still be successfully repeated:
1. Compared to passengers, oncogenic SVs are less likely to create neoantigens (82.2% vs 47.2%, P < 2.2e-16) (Fig. R1c in the "Response letter" docx file).
2. Subclonal SVs are more likely to create neoantigens than clonal SVs (Fig.R1d in the "Response letter" docx file).
3. In breast and ovarian cancer, the differences in GANB between immune-enriched and* *immune-depleted tumors are larger than the differences in TNB (Fig.R1e in the "Response letter" docx file).
4. GANB better predicted the survival of breast cancer than TNB (Fig.R1f in the "Response letter" docx file).
We also agree that repeating the analyses using experimentally determined MHC binding peptides from IEDB would further strengthen our results. However, because most neoantigens are unique to individual cancer patient (Fig. 3A), very few neoantigens analyzed in our work are in IEDB. We therefore could not use the binding peptides from IEDB for independent validation.*

Comment 2: In a similar line, the author's reply to my request to adapt Figure 4A to perform the analysis on cancer type level, does not convince me. They argue that for most genes they have only small number of samples with neo-peptides and neo-epitopes per cancer types and hence the analysis is not feasable on cancer type level. I get this point but it is very risky to compute the expression ratios pooling a large number of tissues (given that the number and tissue composition of neo-peptides and neo-epitopes largely differs, which can easily create confounders). At least they should control for tissue composition when computing the ratios (ie subsample neo-epitopes / -peptides in order to have expression values from the exact same composition of tissues in the numerator and denominator).

*Response: Thanks for pointing out this problem. To address the potential confounding problem by the tissue composition, we first normalized the gene expression within each cancer type and converted the gene expression values to Z-scores. The Z-scores are then directly comparable between different cancer types. Then, we performed the similar analysis with Z-scores (taking differences between Z-scores instead of taking ratios between expressions). Similar to Fig. 4A., we found that the samples bearing neo-epitopes tended to have smaller Z-scores than those bearing neo-peptides (Fig. R2a in the "Response letter" docx file). We added this analysis to the revised manuscript (Supplementary Fig. 6b).
Further, we also categorized the data by cancer type and re-calculated the ratios in Fig. 4A. Genes including SLC25A21, CTNNA3, ARHGAF3, QSER1, CADPS2 and YWHAE were excluded from this analysis because they had no neo-epitope and neo-peptide from the same cancer type. We found that, except from LRP1B in lung squamous carcinoma and MACROD2 in breast adenocarcinoma, all other genes showed the same trends with Fig.4A (Fig. R2b in the "Response letter" docx file). Most genes (81.8%) harboring neo-epitopes were down-regulated compared to those harboring neo-peptides.*

**Third round of review**

**Reviewer 1**

All my concerns have been addressed.
